# Demystifying Nanotherapeutic Modalities: Unravelling the Mysteries from Antibiofilm Mechanisms to Regenerative and Clinical Translation

**DOI:** 10.3390/biomedicines14092030

**Published:** 2026-09-09

**Authors:** Muhammad Umar Javed, Changying Zhang, Zhengwei Huang, Bing Guo

**Affiliations:** 1 School of Science, College of Frontier Sciences, Harbin Institute of Technology, Shenzhen 518055, China; 2 State Key Laboratory of Bioactive Molecules and Druggability Assessment, Guangdong Basic Research Centre of Excellence for Natural Bioactive Molecules and Discovery of Innovative Drugs, College of Pharmacy, Jinan University, Guangzhou 511443, China

**Keywords:** dental infection, nanomaterials, phototherapies, sonodynamic therapy, nanozymes, combinatory therapies, tissue regeneration

## Abstract

Dental infections continue to pose a major health concern worldwide, driven by persistent microbial biofilms, dysregulated host responses, and suboptimal efficacy of conventional antimicrobial therapies. The emergence of nanotechnology has facilitated the development of therapeutic approaches that offer spatiotemporal, minimally invasive, and resistance-preventing alternatives for treating complex dental infections. This review critically assesses novel nanomaterial-based therapies designed to directly eradicate pathogenic microorganisms involved in periodontal disease, dental caries, oral candidiasis and endodontic infections. We first explore the roles of microbial dysbiosis and biofilm-associated pathogenicity as key factors in disease progression, thereby providing a biological rationale for targeted therapies. Particular emphasis is placed on pathogen-directed modalities such as photodynamic, photothermal, sonodynamic, chemodynamic, and nanozyme-based therapies, which allow localized microbial eradication while reducing antibiotic resistance. The design and effectiveness of multifunctional nanomaterials, ranging from bioactive nanoparticles and polymeric hydrogels to microsphere-based delivery systems and implant surface modifications, are analysed in the context of antimicrobial efficacy, biofilm disruption, and compatibility with regenerative processes. Meanwhile, recent advances in integration with tissue regeneration and the potential of artificial intelligence in diagnosis, treatment strategies and materials design are explored as complementary strategies that could improve therapeutic outcomes. By consolidating mechanistic and translational insights, this review highlights how pathogen-targeted nanotherapeutics are revolutionizing the treatment of dental infections with more effective and clinically viable solutions.

## 1. Introduction

Oral health has a significant effect on one’s mental and psychosocial well-being beyond localized tissue damage, as it can affect nutrition, personality, speech, quality of life, and social appearance [1]. Moreover, oral diseases cause secondary disorders and can affect other organs and systems [2]. In the absence of oral hygiene and regular care, the excessive accumulation of microorganisms and their by-products causes inflammation, infections, bleeding, gum swelling, and tooth loss [3]. Oral infections include dental caries, periodontitis, endodontic infections, peri-implantitis, and oral candidiasis [4,5]. Despite advancements in preventive dental care and improved public awareness, the worldwide prevalence of oral infectious ailments continues to rise due to dietary changes, systemic problems, aging populations, and increased retention of natural dentition into the progressive phase [6]. Various mechanistic and epidemiological studies have shown the substantial biomedical significance of chronic oral infections linked with systemic disorders, including diabetes mellitus, rheumatoid arthritis, cardiovascular disease, and adverse pregnancy outcomes [7].

Most dental infections are caused by microbial dysbiosis and the formation of structured biofilms within the oral cavity [8]. The initial substrate for microbial adhesion is provided by the acquired pellicle, resulting from the salivary proteins on tooth and implant surfaces. The resulting colonization leads to diverse and complex biofilm communities embedded within an extracellular polymeric substance (EPS) matrix composed of lipids, polysaccharides, proteins, and nucleic acids. This matrix not only stabilizes microbial architecture but also creates diffusion barriers that limit antimicrobial penetration and protect resident pathogens from host immune surveillance [9,10,11].

*Streptococcus mutans* (*S. mutans*) is the key pathogen implicated in dental infections, which plays a dominant role in caries formation through its aciduric and acidogenic characteristics [12]. These pathogens promote localized enamel demineralization and biofilm cohesion by synthesizing extracellular glucans via glucosyltransferases [13]. A keystone pathogen in periodontitis, Porphyromonas gingivalis (*P. gingivalis*) has a significant impact on microbial ecology and host responses. Through gingipain-mediated proteolysis and complement manipulation, it sustains a chronic inflammatory state conducive to connective tissue breakdown and alveolar bone loss [14,15]. Additionally, other microbes, such as *Candida albicans* (*C. albicans*), display structural elasticity, transitioning between yeast and hyphal forms that promote immune evasion and epithelial invasion, principally in immunocompromised individuals [16,17]. Growing evidence also reveals that intestinal bacteria, such as *Escherichia coli* (*E. coli*), further complicate infection dynamics by disseminating via extraoral pathways. Although *E. coli* is not a predominant oral pathogen, its presence in certain oral microbial communities and its relevance to systemic or extraoral infection pathways provide a broader context for evaluating antimicrobial nanotherapeutics [18].

Biofilm-mediated resistance is the main cause of this pathogenic resilience [19]. Reduced metabolic activity within biofilm cores, efflux pump expression, quorum-sensing signalling, and enzymatic inactivation collectively diminish antibiotic efficacy [20,21]. Therefore, clinical management frequently relies on surgical intervention, drainage, or mechanical debridement, with systemic antibiotics employed as adjuvant therapy [22]. However, prolonged or inappropriate antibiotic usage promotes dysbiosis, secondary fungal overgrowth, and selection of resistant strains [23]. Antimicrobial resistance has been reported in several clinically relevant oral anaerobic species, although the prevalence and resistance profiles vary considerably among bacterial species, geographic regions, antibiotic classes, and clinical populations [24].

Given these limitations and problems, advanced therapeutic modalities based on nanotechnology, materials science, and precision medicine have emerged in recent years. Among these, photodynamic therapy (PDT) [25], chemodynamic therapy (CDT) [26], sonodynamic therapy (SDT) [27], and photothermal therapy (PTT) [28], are promising non-antibiotic techniques for eliminating dental infections through chemical or physical disruption. PDT employs photosensitizers that are stimulated by specific wavelengths to generate reactive oxygen species (ROS). Unlike antibiotics, ROS-mediated killing targets multiple cellular processes, minimising the likelihood of resistance development. Several studies have shown that nanoparticle-encapsulated photosensitizers enhance biofilm penetration and prolong retention within periodontal pockets [29]. CDT has recently gained attention for exploiting the infection microenvironment itself. Fenton or Fenton-like reactions catalyzed by metal-based nanoparticles produce extremely cytotoxic hydroxyl radicals (•OH) by leveraging endogenous hydrogen peroxide and acidic pH within diseased tissues [30]. SDT expands on this paradigm by utilizing ultrasound (US) to stimulate sonosensitizers, permitting deeper tissue penetration than light-based approaches. This is especially beneficial in anatomically complex areas such as extensive periodontal pockets and root canals. Current research has shown that US-responsive nanomaterials can cause ROS production and cavitation effects, efficiently destroying bacterial aggregation embedded in dense matrices [31]. PTT represents another well-established modality utilizing light-absorbing nanomaterials to generate localized hyperthermia [32]. Parallel to stimulus-responsive treatments, nanozyme-based technologies have familiarized catalytic functionality into dental antibacterial approaches [33]. These artificial nanomaterials mimic natural enzyme actions such as oxidase, peroxidase, or catalase. Nanozymes improve biofilm vulnerability and pathogen clearance via ROS generation or degradation of EPS components [34].

The efficiency of these modalities strongly relies on biomaterial design [35]. Polymeric hydrogels offer localized retention and prolonged release, while conforming to uneven periodontal defects [36,37]. Stimuli-responsive nanocarriers allow on-demand stimulation triggered by US, light, or chemical signals [38]. Inorganic nanoparticles provide catalytic efficiency and structural robustness, while implant-surface adjustments integrate antibacterial coatings to prevent early colonization [39]. Notably, these systems can be engineered to respond to particular physicochemical characteristics of infected locations, such as high glutathione levels, acidic pH, or enlarged hydrogen peroxide concentration, thereby optimizing antibacterial accuracy while reducing systemic toxicity [40].

In addition to pathogen elimination, efficient infection control promotes tissue regeneration [41]. While regenerative approaches are not the prime focus of nanotherapeutic strategies, research shows that combining antibacterial nanoplatforms with bioactive scaffolds can promote subsequent endodontic or periodontal regeneration. This combination highlights the importance of therapeutic approaches that balance antibacterial effectiveness with cytocompatibility [42,43,44].

The integration of artificial intelligence (AI) further accelerates advancement in this field. Machine learning (ML) algorithms are increasingly employed to predict nanoparticle-biofilm interactions, optimize photothermal conversion efficiency, and screen candidate materials for catalytic performance. AI-assisted imaging procedures facilitate early diagnosis of lesions and enable immediate analysis of therapeutic results, implying for personalized treatment designs [45,46].

**Scheme 1:**
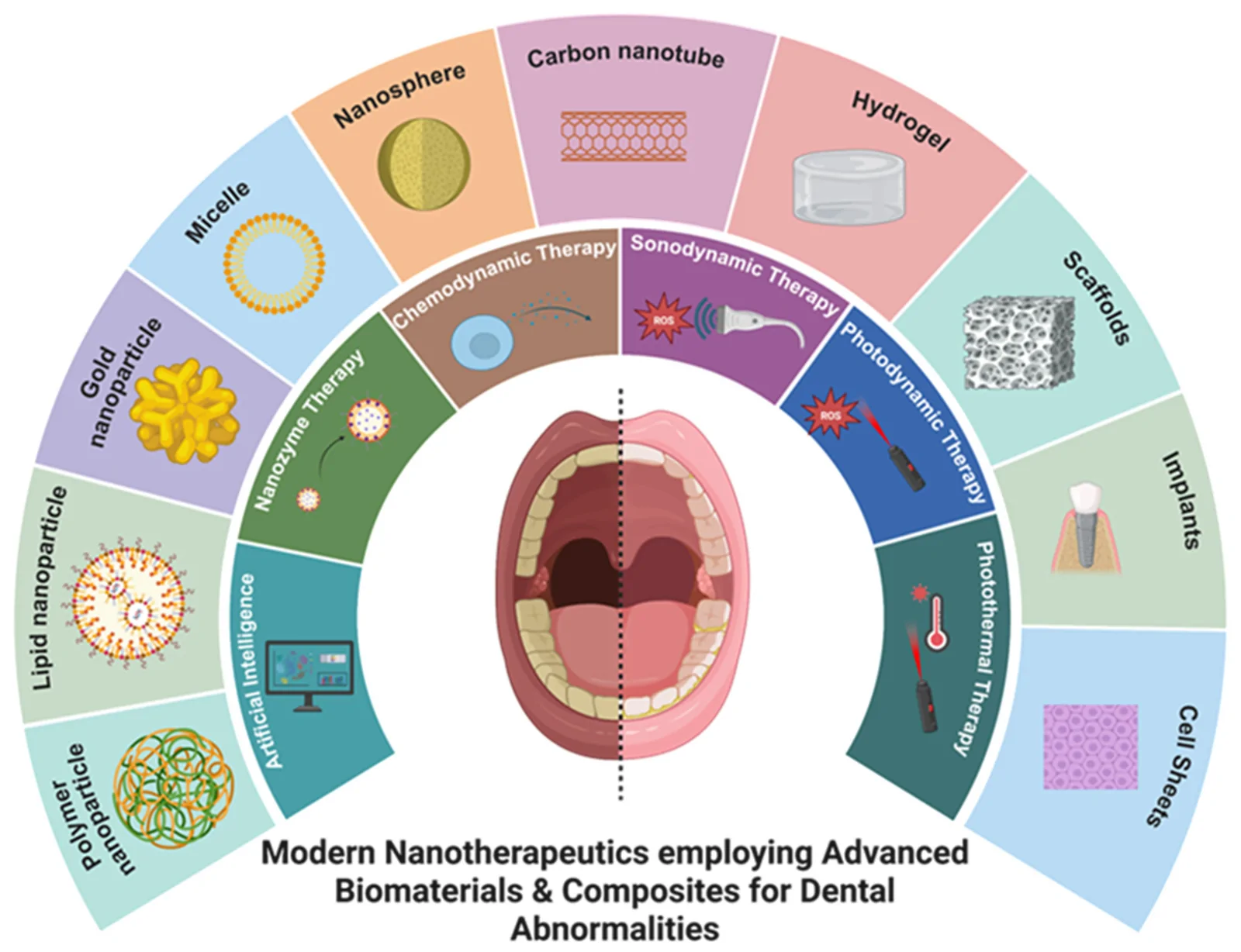
Targeted therapies, along with biomaterial development and dental constructs for dental healthcare management.

Despite significant advancement, existing literature frequently reports a specific modality in isolation [47]. A comprehensive framework that combines modality-specific efficacy with microbial pathogenesis and biomaterial engineering remains limited. This review, therefore, outlines existing nanotherapeutic modalities for dental infections, highlighting biomaterial efficacy and mechanistic rationale across PDT, CDT, SDT, PTT and nanozyme platforms, and translational considerations (Scheme 1). By focusing on resistance-independent microbial control supported by advanced materials design, we aim to outline a comprehensive roadmap for next-generation therapeutic interventions in oral infectious diseases.

## 2. Microbial Etiology and Disease Progression

**Figure 1:**
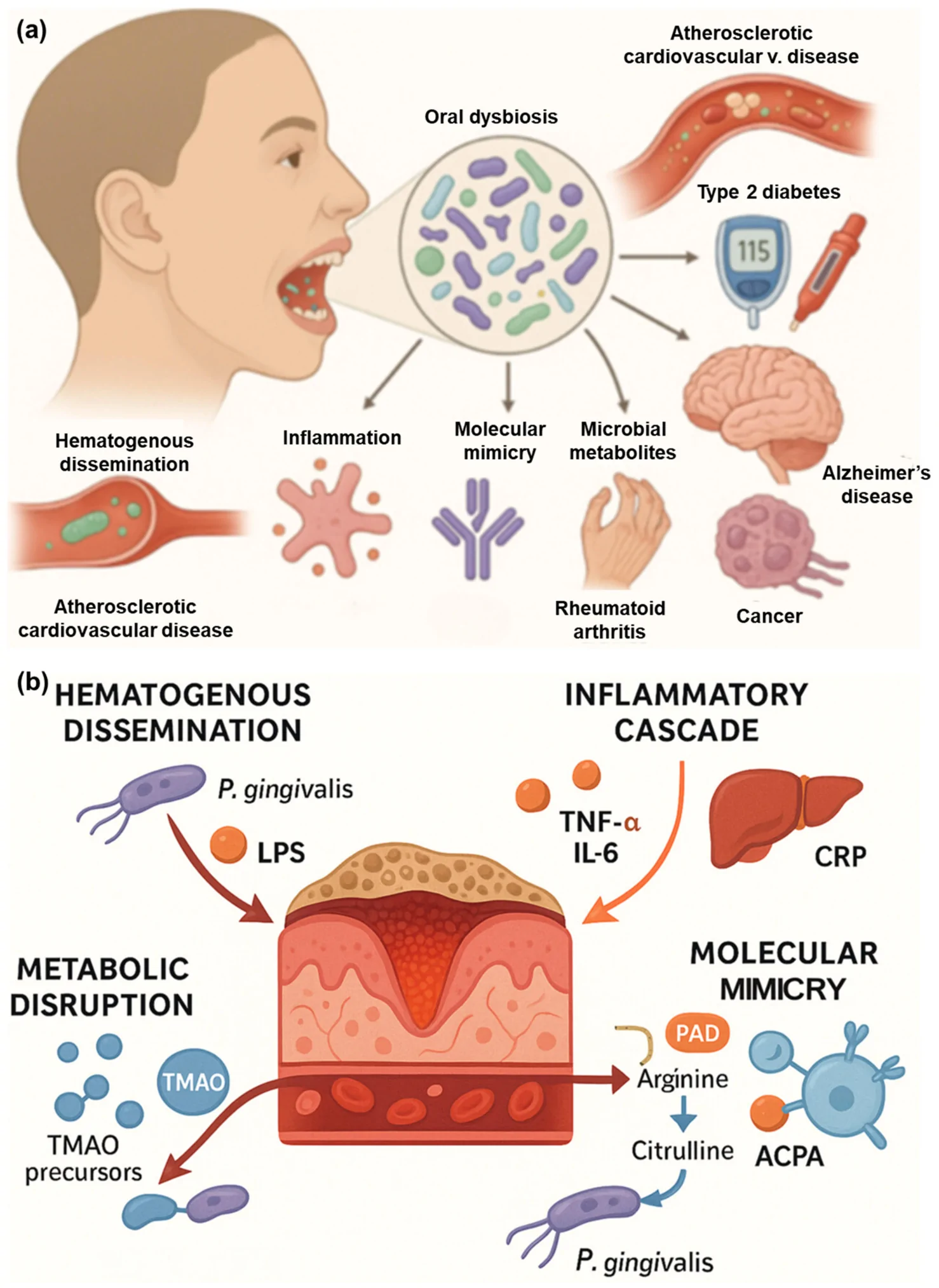
(**a**) Human oral microbiome with a complex ecosystem in health and dysbiosis. (**b**) Key mechanisms connecting oral dysbiosis to systemic disease [48]. Reproduced with permission from ref. [48], Copyright 2026, Taylor & Francis.

The human oral microbiome is an extremely varied ecosystem with significant roles in systemic and oral health. Beyond periodontitis and dental caries, oral dysbiosis has been progressively linked to the development of several non-communicable diseases (Figure 1a). Studies have shown that oral dysbiosis affects systemic health through multiple mechanisms, including chronic systemic inflammation, hematogenous dissemination of oral pathogens and virulence factors (e.g., lipopolysaccharide), microbial metabolic by-products, and molecular mimicry in autoimmune disorders (Figure 1b) [48]. The dynamic microbial ecosystem of the human oral cavity harbours more than 770 bacterial species, along with several fungi and viruses, each causative of oral disease or homeostasis [49,50]. It is essential to understand the mechanisms by which these microbes become pathogenic, and environmental factors inducing susceptibility, to develop targeted interventions [51]. Common oral inhabitants such as *P. gingivalis*, *S. mutans*, *T. denticola*, and *C. albicans* are often implicated in dental pathologies [52]. The conformation of the oral microbiota diverges among individuals due to effects including hygiene, diet, genetic makeup, and environmental exposure. Microbial balance can be disrupted by factors such as antibiotic use, smoking, or systemic infections, leading to dysbiosis and the emergence of pathogenic phenotypes. When commensal microorganisms transition into virulent forms, they evade immune defences and initiate disease processes [53]. The first film on the tooth surface that initiates plaque formation is the acquired pellicle. The attachment of early biofilm colonisers to the pellicle’s proline-rich proteins via surface adhesin receptors comes next. By revealing cryptotopes, this initial binding encourages co-aggregation and permits layer-by-layer plaque development. The local microenvironment becomes increasingly anaerobic due to widespread deposition and layer-by-layer plaque formation, which creates an ideal setting for anaerobic bacterial growth and signifies the progression of gingivitis to periodontitis [54,55]. Multispecies biofilms further complicate antimicrobial treatment because microbial interactions, extracellular polymeric substances, metabolic cooperation, ecological succession, and spatial heterogeneity can alter susceptibility compared with single-species cultures. Consequently, broad-spectrum eradication of oral microorganisms may not necessarily represent an optimal therapeutic outcome. Selective disruption of pathogenic or dysbiotic communities while preserving commensal microorganisms may provide a more desirable strategy for maintaining oral ecological stability. Importantly, selective reduction of pathogenic biofilms should not be equated with preservation of beneficial microorganisms unless this effect has been demonstrated using comprehensive multispecies or microbiome-level analyses [56,57,58,59]. Oral pathogens are fundamental contributors to dental ailments like periodontal disease and dental caries, exhibiting diverse morphologies appropriate to their ecological niches. These pathogens activate host immune responses, stimulating pro-inflammatory mediators such as TNF-α, IL-1, and MMPs, which contribute to connective tissue breakdown and alveolar bone resorption. Among the most prominent are *P. gingivalis*, *A. actinomycetemcomitans*, *T. forsythia*, and *T. denticola*, particularly in the context of periodontitis [60]. Caries development is primarily linked to biofilm-forming, acidogenic species such as *S. mutans*, especially under conditions of poor hygiene and dietary sugar exposure. Mutualism between the host and microbes sustains the homeostatic balance in the oral microbiome. However, a shift toward parasitism underlies disease onset, particularly in caries and inflammatory periodontal conditions [61,62].

The oral microbiome comprises commensal microorganisms, pathobionts, opportunistic pathogens, and disease-associated microbial communities whose interactions can change with environmental conditions and host factors. Certain microorganisms may contribute disproportionately to disease through keystone functions or ecological interactions even when their relative abundance is not dominant [63]. Current data indicate that dominant pathogens by prevalence include herpes viruses (60%), *C. albicans* (22%), *S. mutans* (6%), *P. gingivalis* (5%), and others [60]. *C. albicans* remains the primary fungal agent of oral candidiasis, though related species like *C. glabrata* and *C. dubliniensis* are also implicated. Pathogenesis involves fungal adhesions such as HWP1 and ALS3 binding to epithelial receptors (e.g., E-cadherin, EphA2), enabling antifungal resistance and biofilm formation. Co-aggregation with bacteria like *S. gordonii* further stabilizes biofilms via protein-protein interactions (ALS3-SspB). The hyphal conversion boosts tissue invasion, assisted by thigmotropism and the secretion of degradable enzymes (SAPs, phospholipases). Candidalysin, a hypha-specific toxin, exacerbates host cell damage, while internalization into host cells is mediated by ALS3 and Ssa1 through degradation of intercellular junctions [64]. The oral microbiota’s influence extends beyond local pathology. Microbial dysbiosis has been linked to disease initiation and progression in rheumatoid arthritis (RA). *P. gingivalis* plays a key role in ACPA production in RA by inducing citrullination of host proteins through its peptidylarginine deiminase (PPAD). This effect is further amplified by neutrophil extracellular traps (NETs), generated during infection through PAD4. Dysbiotic patterns characterized by elevated *Veillonella*, *Prevotella*, and *L. salivarius*, along with abridged *Haemophilus* spp., promote Th17-mediated inflammation. Molecular mimicry, such as that between *P. gingivalis* α-enolase and its human counterpart, may further perpetuate autoimmune activation [64].

This interrelation of systemic and oral health highlights the significance of microbial equilibrium in the oral cavity. Microbial translocation between the oral and gut microbiomes supports a bidirectional model, wherein disturbances in one niche may provoke systemic consequences [65]. As evidence accumulates, maintaining oral microbial balance is increasingly recognized as integral to whole-body health. Advancements in this field support a multidisciplinary approach, incorporating evidence-based strategies such as the use of dental probiotics (*S. salivarius*, *Lactobacillus* spp.), xylitol-containing formulations, fluoride-based products, and essential oil mouth rinses [66,67]. Continued exploration of novel oral health products and cross-disciplinary methodologies will be key to enhancing our understanding of the oral microbiota and developing targeted, effective interventions.

## 3. Nanotherapeutic Strategies

Since biofilm formation significantly enhances bacterial resistance to traditional antibiotics, bacterial infections remain a substantial challenge to human health. The misuse and overuse of these antibiotics have further contributed to the prevalence of multidrug-resistant bacteria, rendering many conventional therapies progressively ineffective and raising global health risks. In light of this emerging challenge, the development of innovative antibacterial paradigms has become imperative. Recent developments have explored a range of non-antibiotic therapeutic strategies, including PDT, SDT, CDT, PTT, nanozyme-based interventions, and various combinatorial treatments utilizing various bioactive materials. These emerging modalities, characterized by their targeted action and minimal invasiveness, hold substantial potential for combating bacterial infections, predominantly in dentistry, where diseases like periodontitis, dental caries, and biofilm-mediated resistance remain prevalent and challenging to manage. However, the relative performance of nanotherapeutic modalities is highly dependent on the characteristics of the infection and the local microenvironment rather than on the inherent superiority of a single approach. PDT can provide spatially controlled ROS generation but may be constrained by oxygen availability and photosensitizer penetration in mature biofilms. PTT offers rapid and localized thermal effects and is less dependent on oxygen, although excessive heating may damage surrounding tissues and its effectiveness can decrease with limited heat penetration. SDT provides deeper stimulus delivery through ultrasound and may therefore be advantageous for less accessible infection sites, but its performance is strongly influenced by ultrasound parameters and the local tissue environment. CDT and nanozyme-based systems can generate ROS through catalytic reactions and may operate without external light or ultrasound, although their activity depends on factors such as local pH, substrate availability, and catalytic stability. Importantly, combining complementary modalities can overcome some of these limitations by integrating different antimicrobial mechanisms and improving treatment efficiency. Thus, the selection of a nanotherapeutic strategy should be guided by the infection site, biofilm characteristics, local microenvironment, required penetration depth, activation conditions, and safety considerations rather than by antimicrobial potency alone.

### 3.1. Photothermal Therapy

PTT is gaining considerable attention in contemporary dentistry as an innovative approach for managing bacterial infections [68]. This technique harnesses the heat generated by light-absorbing photothermal agents (PTAs), particularly those responsive to NIR, to induce localized hyperthermia. Upon NIR exposure, PTAs efficiently convert light energy into heat, which can compromise bacterial cell membranes, denature critical proteins, and ultimately lead to irreversible microbial inactivation [69,70]. The success of PTT hinges on the characteristics of the PTAs employed; ideally, these should demonstrate high photothermal conversion efficiency, biocompatibility, thermal resilience, and cost-effectiveness [71]. Various classes of PTAs, including noble metal nanoparticles, conductive polymers, organic chromophores, and metal oxides, are currently under investigation. NIR wavelengths ranging from 700 to 950 nm are especially advantageous due to their superior tissue penetration and minimal absorption by endogenous biomolecules such as water and haemoglobin. NIR wavelengths are widely investigated for photothermal applications because selected spectral regions can provide relatively favorable tissue penetration compared with shorter wavelengths; however, optical attenuation remains wavelength- and tissue-dependent and is influenced by endogenous chromophores, including haemoglobin and water [72,73]. Unlike conventional antibiotics, PTT delivers rapid, site-specific antimicrobial action with a broad spectrum of activity, including efficacy against biofilm-embedded and drug-resistant microorganisms [74,75]. Its integration into procedures like root canal disinfection underscores its potential as a minimally invasive and resistance-free alternative to standard antimicrobial therapies, particularly for targeting pathogens such as *S. mutans* [76,77,78]. With its precise action, speed, and low potential for resistance development, PTT represents a forward-looking modality in the advancement of dental infection control.

Li et al. [79] introduced a multifunctional foam-based dental brace embedded with a magnesium-organic framework composite (Mg-MOF@PDA@CaP, abbreviated as MPC) designed for dual-purpose intervention, managing dental caries and promoting enamel remineralization. This cognitive platform responds selectively to acidic microenvironments by releasing therapeutic agents in a controlled manner, which is a characteristic of cariogenic biofilms. Under low pH conditions, MPC liberated a combination of bioactive molecules, gallic acid, a naturally derived antimicrobial compound, along with Mg^2+^, Ca^2+^, and PO_4_^3−^. These mediators worked synergistically to dismantle biofilm architecture, neutralize local acidity, and facilitate mineral deposition at demineralized enamel sites.

**Figure 2:**
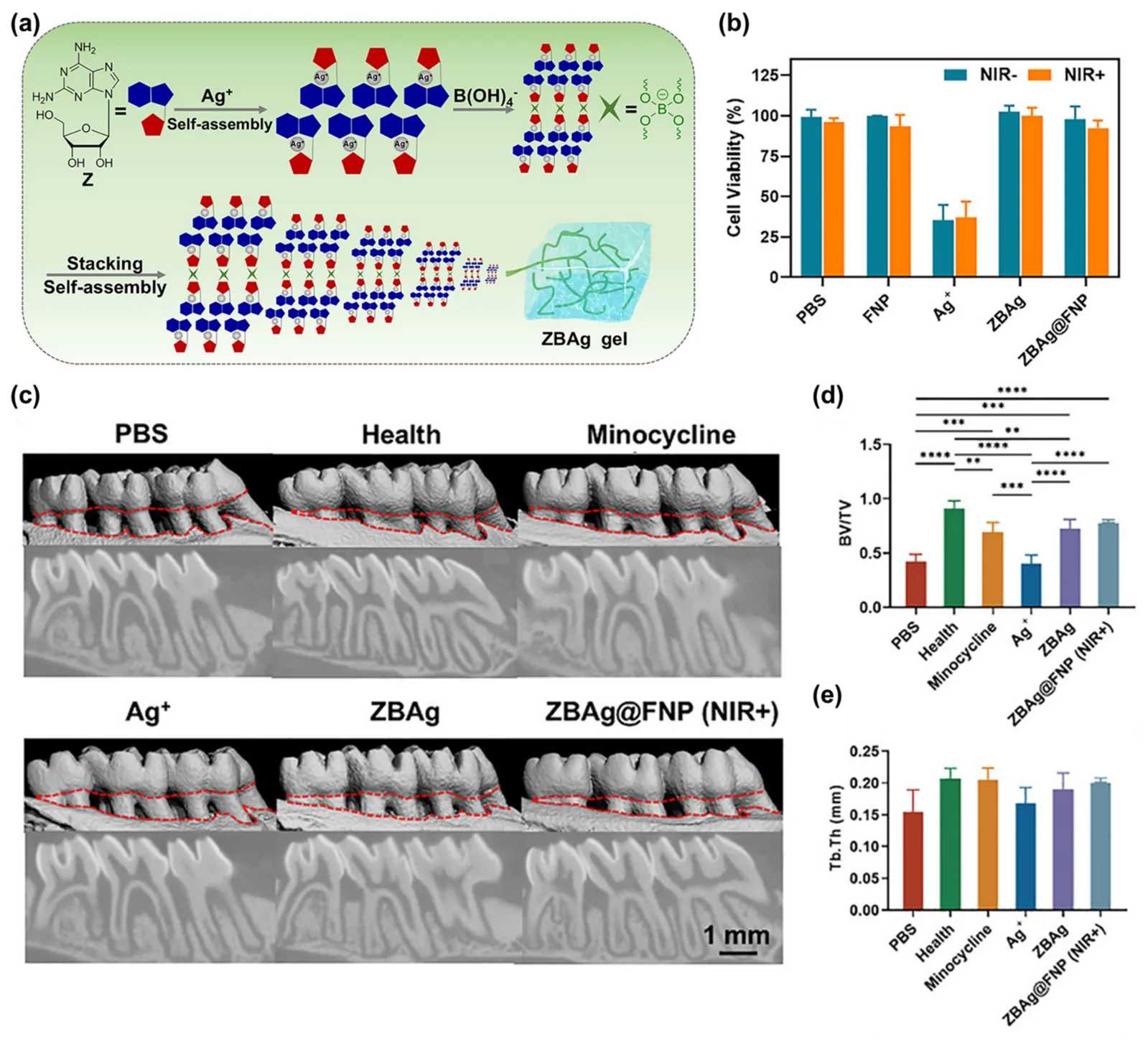
(**a**) Diagrammatic representation of the synthesis of ZBAg hydrogel. (**b**) Cytotoxicity of the L929 cells treated with PBS, FNP, Ag^+^, ZBAg and ZBAg@FNP hydrogels (n = 3). (**c**) Micro-CT analysis representing 3D reconstruction images of the maxillary molars under various treatments (red lines indicative of the ABC-CEJ spatial relationship, scale bar: 1 mm). (**d**) Quantitative examination of BV/TV. (**e**) Quantitative examination of Tb.Th. ** *p* < 0.01, *** *p* < 0.001, **** *p* < 0.0001 [80]. Reproduced with permission from ref. [80], Copyright 2025, WILEY.

Wen et al. [80] developed two organic small-molecule photothermal agents, BF and BCl, featuring strong near-infrared (NIR) absorption through a donor-acceptor molecular design strategy. To construct a multifunctional therapeutic platform, 2-aminoadenosine (Z), a nucleoside analogue containing dual amino groups and multiple hydrogen-bonding sites, was assembled into a supramolecular hydrogel (ZBAg) through Ag^+^-mediated base pairing and dynamic boronate ester interactions. Encapsulation of the photothermal nanoparticle (FNP) within this matrix yielded the ZBAg@FNP hydrogel, which exhibited favorable mechanical strength and efficient photothermal conversion (Figure 2a). Given the importance of biodegradability and biosafety for clinical translation, the in vivo degradation behavior and biocompatibility of the hydrogel were evaluated. Following subcutaneous injection into BALB/c mice, ZBAg and ZBAg@FNP remained at the injection site for approximately 7 and 9 days, respectively, whereas PBS was rapidly cleared. In vitro CCK-8 assays using L929 cells demonstrated negligible cytotoxicity for FNP, ZBAg, and ZBAg@FNP, while free Ag^+^ reduced cell viability to below 50%, indicating that the hydrogel matrix effectively mitigated Ag^+^ toxicity through controlled ion release (Figure 2b). These findings confirmed the excellent biocompatibility and sustained-release characteristics of the ZBAg@FNP system. Antibacterial evaluation revealed that Ag^+^-containing groups exhibited inhibitory activity under both light and dark conditions, while NIR-triggered photothermal heating provided an additional bactericidal effect. Notably, ZBAg@FNP combined with NIR irradiation displayed the strongest antibacterial performance against planktonic bacteria, highlighting the synergistic action between localized PTT and sustained Ag^+^ release.

The therapeutic efficacy of this platform was further investigated in a rat periodontitis model. Micro-CT analysis showed extensive alveolar bone resorption in the PBS-treated group, extending from the second molar to adjacent teeth. Although both minocycline and ZBAg treatments partially preserved alveolar bone, the ZBAg@FNP-treated group exhibited minimal radiographic evidence of bone loss, demonstrating superior therapeutic efficacy (Figure 2c). Quantitative assessment of the alveolar bone crest-to-cementoenamel junction (ABC-CEJ) distance further confirmed markedly reduced bone resorption in the ZBAg@FNP + NIR group. Consistent trends were observed for bone volume to total volume ratio (BV/TV) and trabecular thickness (Tb.Th), where treatment effectively suppressed bone loss while improving trabecular architecture (Figure 2d,e). Histological examination by H&E staining also revealed a substantial reduction in inflammatory cell infiltration following treatment. Overall, the ZBAg@FNP hydrogel represents an effective antibiofilm platform that combines NIR-responsive photothermal therapy with controlled Ag^+^ release. By integrating localized bacterial eradication with prolonged antimicrobial activity, this strategy offers a promising approach for periodontal infection management. It provides valuable insights for the design of next-generation photothermal antibacterial biomaterials. Collectively, PTT has demonstrated considerable potential for localized elimination of oral pathogens through controlled photothermal effects, with evidence extending from in vitro experiments to selected in vivo models. Nevertheless, these results should not yet be interpreted as established clinical efficacy, as further studies are needed to determine the optimal thermal window, treatment penetration, long-term tissue safety, and therapeutic performance under clinically relevant conditions.

### 3.2. Photodynamic Therapy

In recent years, PDT has emerged as a compelling antimicrobial approach, gaining attention for its noninvasive nature, ability to penetrate deep tissues, and lack of association with drug resistance [81,82]. Among various PDT modalities, those employing fluorescent molecules have demonstrated particular promise, offered high spatiotemporal precision, and enabled simultaneous diagnostic and therapeutic functions [83]. In the context of oral healthcare, PDT is being actively explored for its potential in managing dental infections and facilitating tooth whitening procedures. This approach has shown efficacy against a wide range of oral pathogens implicated in conditions such as periodontitis, dental caries, and endodontic infections [84,85]. PDT operates through the activation of photosensitizers (PSs) by specific wavelengths of light, leading to the generation of ROS. These ROS exert oxidative stress on microbial cells by damaging vital biomolecules such as nucleic acids, proteins, and lipids, thereby triggering irreversible cell death. PDT generally proceeds through two principal photochemical pathways. In the Type I pathway, photoexcited sensitizers participate in electron- or hydrogen-transfer reactions that generate reactive radical species, whereas the Type II pathway involves energy transfer from the excited sensitizer to molecular oxygen to produce singlet oxygen. Both pathways can contribute to antimicrobial activity, although their relative contribution depends on the photosensitizer, oxygen availability, irradiation conditions, and local microenvironment. Oxygen depletion within mature biofilms can therefore restrict PDT performance, particularly when treatment relies predominantly on Type II photochemistry [86,87]. Notably, PDT offers several advantages over traditional antimicrobial methods, including minimal tissue damage, low systemic toxicity, and reduced likelihood of developing antibiotic resistance.

Zhang et al. [88] developed a dual-functional photodynamic dental therapy approach aimed at both tooth whitening and biofilm removal, utilizing a zwitterion-functionalized porphyrin as the PS. Even though the system showed significant therapeutic results, its practical implementation was limited by the requirement for prolonged incubation, high PS concentrations, and extended light exposure, all of which could compromise clinical feasibility and patient comfort. Furthermore, conventional PSs tend to exhibit reduced ROS production at high concentrations or in aggregated forms, limiting their efficacy even at higher concentrations. To cope with these challenges, materials exhibiting aggregation-induced emission (AIE) have attracted considerable interest. These AIE-based systems provide notable advantages such as enhanced ROS generation and strong photostability in their aggregated form, rendering them suitable for microbial inactivation and therapeutic applications, including in dental procedures. Tang and colleagues first introduced the concept of aggregation-induced emission (AIE) and elucidated its underlying mechanism. Their pioneering research laid the foundation for the application of AIE-type PSs in both bacterial imaging and antimicrobial therapy. This invention has significantly improved the therapeutic efficacy of photodynamic strategies. AIE luminogens (AIEgens) display a distinct ability to generate ROS more efficiently upon aggregation, a property that has catalyzed rapid progress in their use for diagnostic and therapeutic purposes [89]. These luminogens not only provide high fluorescence intensity for imaging applications but also excel in selective microbial targeting and inactivation. Their combined photophysical and biological properties make AIEgens a promising and versatile platform for advancing antimicrobial treatments [90].

**Figure 3:**
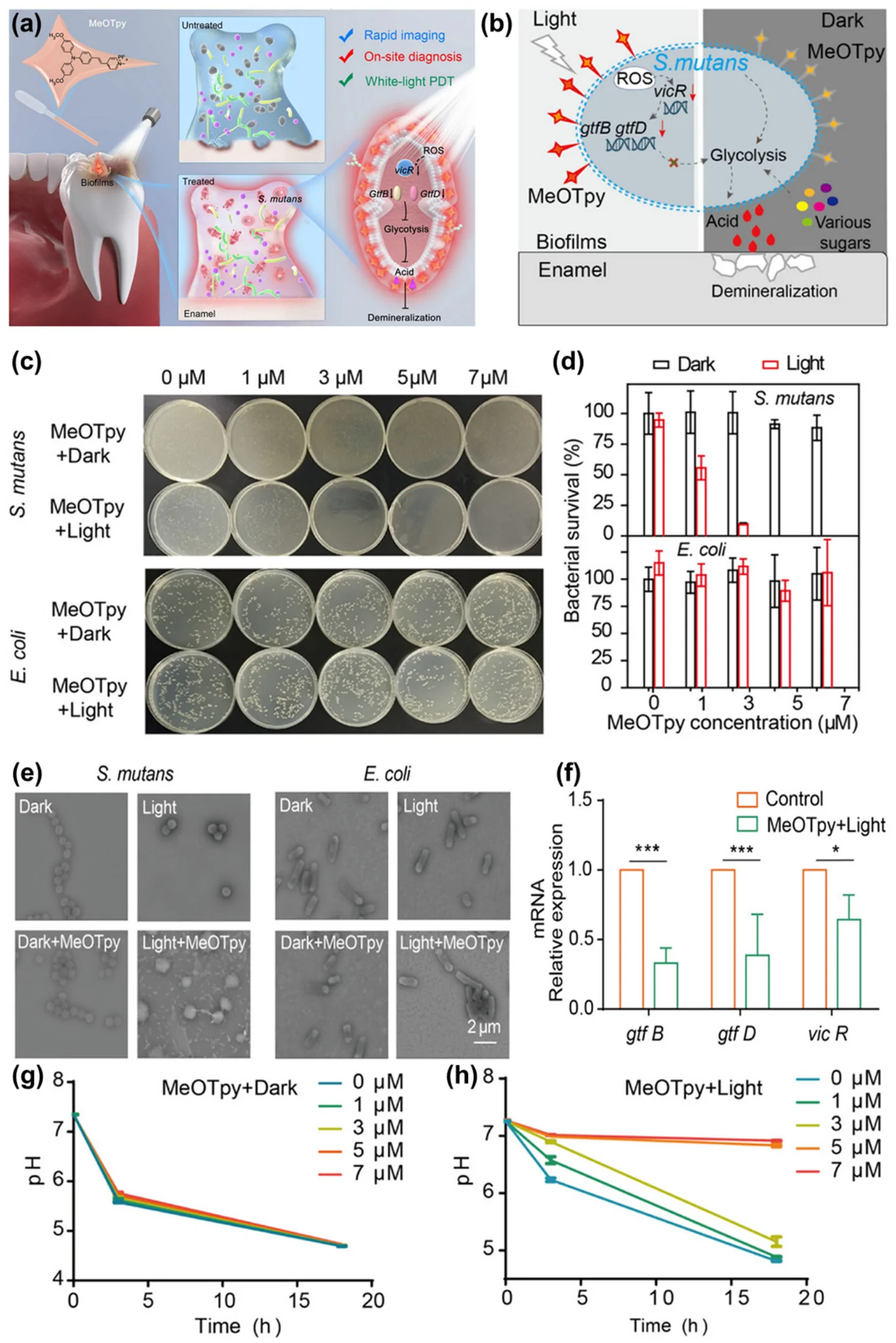
(**a**) Schematic illustration of MeOTpy’s aggregation-induced photosensitivity enabling targeted identification and photodynamic inactivation of cariogenic bacteria. (**b**) Diagram demonstrating the process by which MeOTpy mitigates enamel demineralization under white light exposure. (**c**) Quantitative assessment of bacterial colony formation following treatment with MeOTpy under either white light irradiation or dark conditions for 15 min. (**d**) Comparative survival rates of *S. mutans* and *E. coli* post-treatment. (**e**) SEM images showing morphological changes in bacterial cells treated with 7 µM MeOTpy, with and without white light exposure. (**f**) Relative expression levels of gtfB, gtfC, and vicR genes in *S. mutans*. (**g**,**h**) Acidity assays tracking pH variation in *S. mutans* cultured in BHI medium supplemented with 1% sucrose (initial pH 7.35) at 37 °C, treated with various concentrations of MeOTpy under either dark or illuminated conditions for 15 min. * *p* < 0.05, *** *p* < 0.001 [91]. Reproduced with permission from ref. [91], Copyright 2025, WILEY.

Zhang et al. [91] designed a novel AIE-active molecule, MeOTpy, specifically engineered for theranostic applications in dental caries management (Figure 3a). This compound exhibited aggregation-induced emission properties and showed high selectivity toward gram-positive bacteria. Upon binding to cariogenic strains, MeOTpy emits strong fluorescence, enabling precise visualization, and concurrently generates ROS under white light irradiation. Fluorescence imaging of decayed human teeth treated with MeOTpy confirmed its diagnostic potential in detecting carious lesions. To evaluate its therapeutic efficacy, ex vivo biofilms collected from children with severe early childhood caries (SECC) were cultured and subjected to photodynamic inhibition using MeOTpy. Additionally, a rat pup caries model was employed to assess both the compound’s preventive impact on disease progression and its influence on the composition of the oral microbiota. Interestingly, MeOTpy substantially decreased acid production by *S. mutans*, although MeOTpy exhibited limited direct bactericidal activity at the tested concentration, while producing a pronounced reduction in acidogenesis, biofilm-associated behavior, and the expression of virulence-related genes. These findings suggest that its primary effect under the reported conditions may involve attenuation of cariogenic activity and virulence rather than extensive bacterial killing. Quantitative PCR analysis revealed that MeOTpy suppressed biofilm formation and acidogenicity by inhibiting key caries-associated virulence genes, gtfB, gtfC, and vicR (Figure 3b). Notably, these effects sustained even under acidic conditions (pH 4.5), demonstrating functional stability of MeOTpy within cariogenic microenvironments.

To assess bacterial selectivity, colony-forming unit assays were performed. Both *E. coli* and *S. mutans* showed normal proliferation after dark incubation with MeOTpy (0–7 µM), verifying its lack of dark cytotoxicity (Figure 3c,d). However, under white light irradiation (10 mW cm^−2^ for 15 min), a near-complete eradication of *S. mutans* was achieved at 7 µM, while *E. coli* remained largely unaffected. These results validated MeOTpy’s selective photodynamic activity toward gram-positive bacteria. Further morphological analysis using SEM showed clear structural damage in *S. mutans* following light-activated treatment, whereas *E. coli* retained its integrity under all test conditions (Figure 3e), providing direct visual confirmation of targeted bacterial disruption. The compound’s effect on bacterial acidogenesis was assessed through pH monitoring assays. In the absence of light, MeOTpy did not alter the pH decline associated with acid production by *S. mutans* (Figure 3g). However, Glycolytic pH drop assays corroborated a concentration-dependent suppression of acidification under illuminated conditions (Figure 3h). This study highlights MeOTpy as a promising dual-purpose noninvasive caries detection and treatment agent. Its high fluorescence allows rapid and precise identification of lesions, whereas its ROS-driven photodynamic activity targets gram-positive pathogens selectively, without damaging useful microbes. Animal model findings confirm its capability to reduce the progression of the disease without affecting the balance of the microbes, unlike traditional antimicrobials. Altogether, this AIE-based system provides a potential, antibiotic-free approach that combines accurate diagnosis with targeted therapy for dental caries. Overall, the available evidence highlights the promising antimicrobial potential of PDT, particularly against oral pathogens and biofilms; however, most findings remain based on in vitro or laboratory-scale models. Although emerging in vivo studies provide further support, the clinical relevance of PDT requires validation in physiologically representative models and well-designed human studies, particularly with respect to treatment efficacy, tissue safety, and the influence of oxygen availability.

The performance of conventional PDT may be limited in mature periodontal and peri-implant biofilms, where oxygen availability can be spatially heterogeneous and locally restricted. Because many photosensitizers primarily depend on oxygen-mediated ROS generation, hypoxic regions may reduce antimicrobial efficiency. In addition, the complex architecture and microbial diversity of mature biofilms can restrict photosensitizer penetration and create populations with different metabolic states and treatment susceptibilities. Potential strategies to address these challenges include the development of oxygen-generating nanoplatforms, photosensitizers capable of type-I photochemical reactions, improved penetration into the biofilm matrix, and combination therapies that target multiple microbial survival mechanisms.

### 3.3. Sonodynamic Therapy

Implant-associated bacterial infections pose a formidable challenge in clinical practice. The risk of secondary bacterial infection is significant after prolonged use of dental implants and related bone implants [92]. The antibiotic resistance and immune evasion exhibited by bacterial biofilms contribute to the persistent presence and recurrent nature of such infections, challenging their eradication [93]. External field stimulation-based treatment of biofilm recurrence avoids the risks and patient pain associated with surgery. Consequently, there is an urgent need to develop responsive therapeutic modalities that can effectively disrupt biofilm infections [94]. US has significant potential in unblocking the treatment of deep bone infections. Furthermore, US-responsive therapy can noninvasively treat secondary infections for all periods [95]. SDT strategies are promising for secondary biofilm infections by nonsurgical therapy. SDT involves the generation of toxic ROS by US stimulation of sonosensitizers. Elevated levels of ROS can disrupt bacterial protein leakage and DNA to kill bacteria [96]. However, the major limitation of SDT lies in the suboptimal sonodynamic effect of sonosensitizers and loading of nano-sonosensitizers on implants, so it is imperative to develop efficient film-style sonosensitizers. The existing defective design and band gap engineering fall into modifying the intrinsic electronic states. Although they can somewhat enhance the SDT performance, there has been no qualitative breakthrough [97].

Guan et al. [98] employed plasma-enhanced chemical vapor deposition (PECVD) to fabricate TiO_2_/Ti_2_O_3_/VG (HTO/VG) metainterfacial heterostructure films. Upon receiving US waves, the “honeycomb-like” porous structure served as an acoustic-to-thermal conversion platform. The SDT effect initially disrupted the biofilms, killing some bacteria and causing a loss of “connection” between residual bacteria. In such a scenario, the extraordinary sonodynamics continuously attacked the residual bacteria through the generation of ROS. The synergistic sonodynamic/mild sonothermal therapy of HTO/VG eliminated secondary biofilm infections, and the osseointegration ability significantly improved after the biofilm.

**Figure 4:**
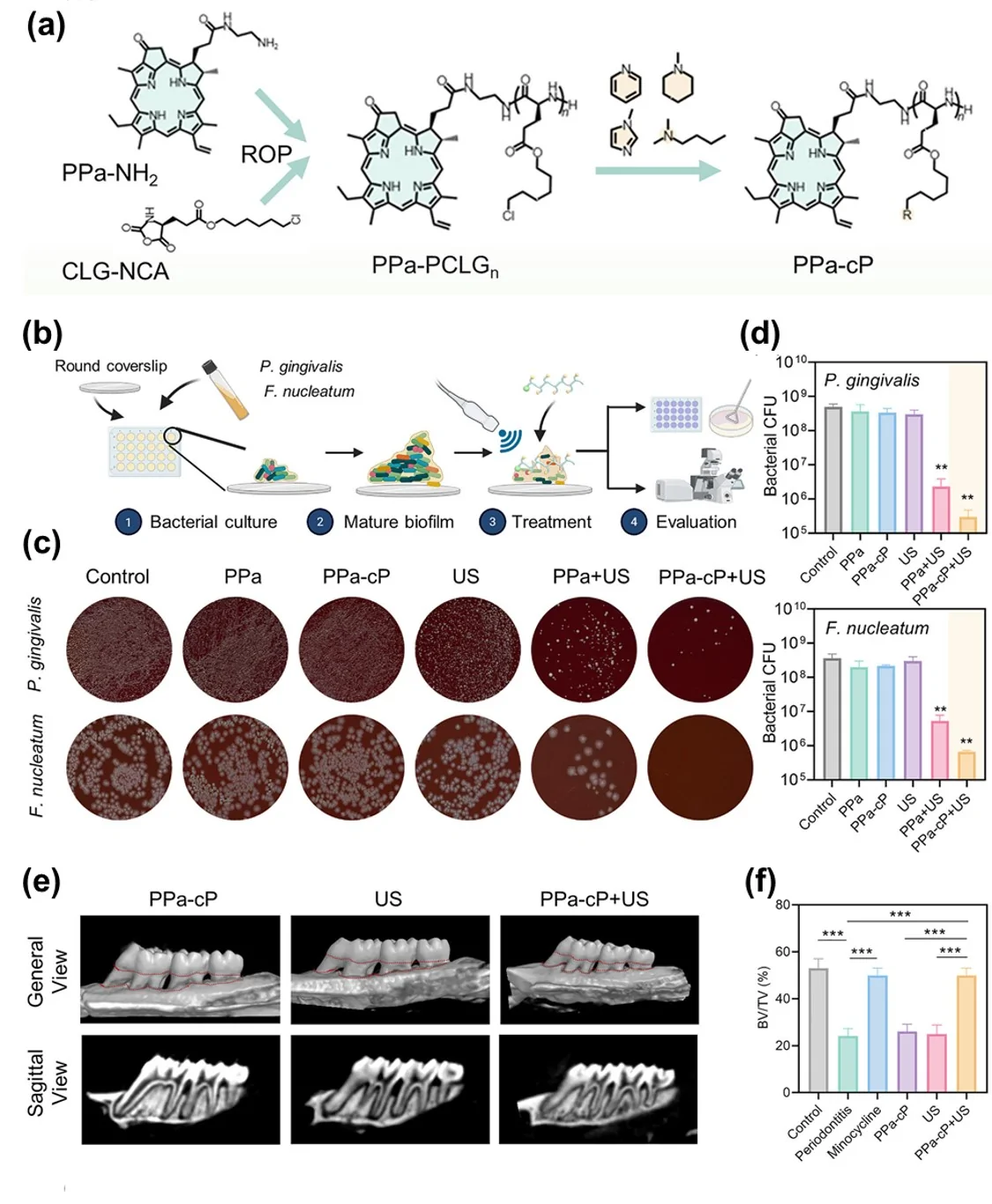
(**a**) Synthesis of the PPa-cationic polypeptide conjugate (PPa-cP). (**b**) Schematic illustration of the experimental protocols for the formation and treatment of biofilms. (**c**) Images of *P. gingivalis* and *F. nucleatum* biofilm colonies treated with PPa-cP + US. (**d**) Corresponding quantitative CFU enumeration. (**e**) Micro-CT analysis of reconstructions of alveolar bone (red lines indicative of the CEJ-ABC distance). (**f**) Quantitative analysis of BV/TV. ** *p* < 0.01, *** *p* < 0.001 [99]. Reproduced with permission from ref. [99], Copyright 2026, WILEY.

Ou et al. [99] developed a cationic polypeptide-conjugated sonosensitizer (PPa-cP) to improve biofilm penetration and enhance the antibacterial efficacy of SDT against periodontal pathogens. The conjugate was prepared through ring-opening polymerization initiated by an amine-functionalized porphyrin derivative (PPa-NH^2^), followed by quaternization to introduce cationic functionalities (Figure 4a). Compared with free PPa, PPa-cP exhibited stronger affinity toward negatively charged bacterial biofilms, enabling improved accumulation within the biofilm matrix. Upon US activation, the generated ROS disrupted bacterial membrane integrity, resulting in efficient biofilm elimination. The antibiofilm performance of PPa-cP was evaluated using *P. gingivalis* and *F. nucleatum*, two representative periodontal pathogens. The experimental workflow for biofilm formation and treatment is illustrated in Figure 4b. Colony-forming unit (CFU) analysis demonstrated that PPa-cP alone exerted minimal antibacterial activity, whereas US-activated PPa-cP significantly reduced bacterial viability. Specifically, SDT treatment decreased *P. gingivalis* biofilm colonies by 3.2 log units (99.9%) and *F. nucleatum* biofilm colonies by 2.74 log units (99.8%) (Figure 4c,d). The greater susceptibility of *P. gingivalis* may be related to its anaerobic metabolic pathways, which are particularly vulnerable to ROS-mediated damage.

Encouraged by its potent antibacterial activity and favorable cytocompatibility, the therapeutic efficacy of PPa-cP was further assessed in a rat model of chronic periodontitis. Periodontal disease was induced by ligation combined with local bacterial inoculation around the maxillary first molar, followed by local administration of PBS or PPa-cP and subsequent US irradiation for 5 min. Micro-CT analysis revealed severe alveolar bone destruction in the untreated periodontitis group, particularly at the root and furcation regions, confirming successful disease establishment (Figure 4e). After two weeks of treatment, the PPa-cP + US group exhibited substantially reduced bone resorption. Quantitative measurements showed that the CEJ-ABC distance decreased to 0.366 mm, closely matching the minocycline-treated group (0.371 mm) and approaching the healthy control value (0.279 mm). In comparison, larger CEJ-ABC distances were observed in the periodontitis (0.775 mm), US (0.752 mm), and PPa-cP (0.718 mm) groups. Consistent with these findings, bone morphometric analysis revealed significant increases in BV/TV and Tb.Th, together with reduced Tb.Sp, in both the minocycline and PPa-cP + US groups (Figure 4f). These results demonstrate that SDT mediated by PPa-cP effectively suppresses periodontal infection, preserves alveolar bone integrity, and restores bone quality to near-normal levels. Taken together, SDT represents a promising approach for treating oral infections, particularly where deeper stimulus penetration may offer an advantage over light-based therapies. Nevertheless, available evidence is predominantly derived from in vitro and early-stage preclinical studies. Further animal and clinical investigations are needed to establish appropriate ultrasound parameters, therapeutic efficacy, tissue compatibility, and long-term safety under clinically relevant conditions.

The therapeutic performance of SDT is strongly influenced by the acoustic conditions used to activate the sonosensitizer. Ultrasound frequency, intensity, duty cycle, exposure duration, and cavitation behavior can affect acoustic energy deposition, ROS generation, treatment depth, and tissue response. Consequently, antimicrobial outcomes obtained under different acoustic conditions cannot be directly compared without considering these parameters. From a clinical perspective, an additional challenge is the controlled delivery of sufficient acoustic energy to anatomically confined sites, including periodontal pockets, root canals, and implant-associated tissues, while minimizing unintended tissue effects. Systematic reporting and optimization of acoustic parameters will therefore be important for establishing reproducible and clinically relevant SDT protocols.

### 3.4. Chemodynamic Therapy

CDT has emerged as a promising antibacterial strategy, particularly effective against biofilm-associated infections such as dental caries and periodontal diseases [100]. CDT utilizes Fenton or Fenton-like reactions to convert endogenous H_2_O_2_, abundant in infected tissues, into highly reactive •OH, which disrupts bacterial membranes and degrades biofilm matrix components like extracellular DNA [101]. This mechanism not only facilitates bacterial eradication but also enhances drug penetration into deep biofilm layers. Dental biofilms, which are notoriously resistant to antibiotics due to their protective EPS, present a microenvironment rich in H_2_O_2_, acidic pH, and GSH, conditions that closely resemble tumor microenvironments and support CDT activation [102]. However, limitations such as insufficient H_2_O_2_ levels, near-neutral pH, and GSH overexpression at infection sites can impair CDT efficacy. To overcome these barriers, engineered nanoplatforms have been developed that enhance H_2_O_2_ supply, promote redox cycling, and deplete intracellular antioxidants. By targeting the unique microenvironment of oral biofilms, CDT offers a non-antibiotic approach with both anti-biofilm and anti-inflammatory potential, making it a compelling strategy for managing persistent bacterial infections in the oral cavity [103].

Jia et al. [104] designed a pH-responsive nanoplatform to enhance CDT by integrating controlled H_2_O_2_ and NO release for effective biofilm eradication. The system comprised a CaO_2_ core coated with HKUST-1, a copper-based MOF, and surface-loaded l-arginine. Under acidic biofilm conditions, HKUST-1 degraded, releasing Cu^2+^ and l-arginine, while exposing CaO_2_ to generate H_2_O_2_. Cu^2+^ catalyzed the Fenton-like conversion of H_2_O_2_ into •OH and •O_2_^−^, while l-arginine yielded NO. These species further reacted to form ONOO^−^, a potent oxidant. Simultaneously, Cu^2+^ depletes intracellular GSH, amplifying oxidative damage. This increased penetration of reactive species into the biofilm was due to NO-induced disruption of the biofilm matrix. The system was highly effective against in vitro MRSA and *P. aeruginosa* biofilms, therapeutic in an in vivo wound model, and demonstrated a synergistic effect on both biofilm disruption and bacterial killing.

**Figure 5:**
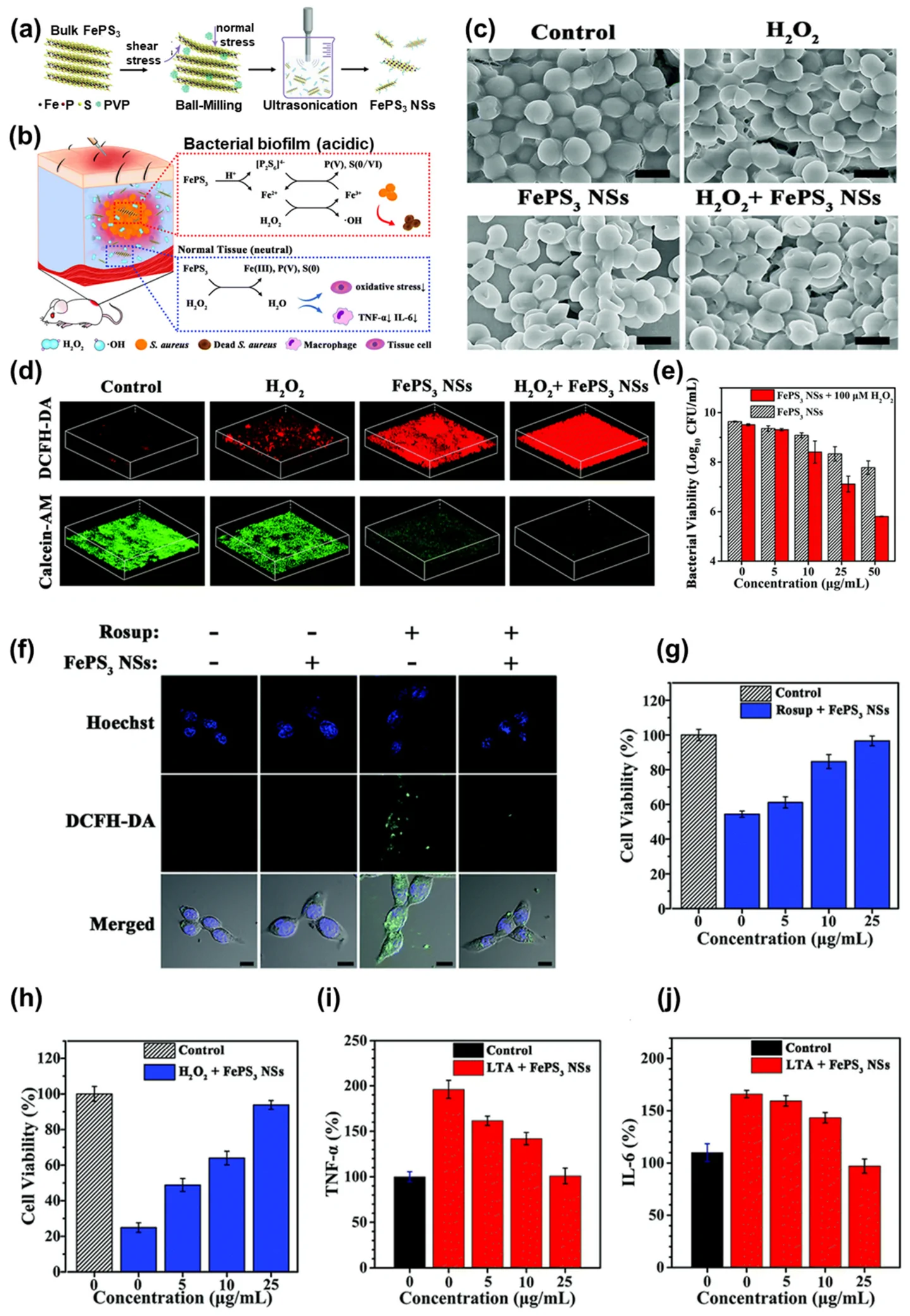
(**a**) Illustration of the synthesis process for FePS_3_ NSs using ball-milling followed by US exfoliation. (**b**) Functional depiction showing that FePS_3_ NSs possess dual activity. (**c**) SEM images of *S. aureus* biofilms following treatment with saline, H_2_O_2_, FePS_3_ NSs, and a combination of FePS_3_ NSs with H_2_O_2_. (**d**) 3D CLSM images of *S. aureus* biofilms subjected to saline, H_2_O_2_, FePS_3_ NSs, and FePS_3_ NSs with μM H_2_O_2_. (**e**) Quantitative CFU analysis of bacterial populations treated with varying doses of FePS_3_ NSs, both in the presence and absence of H_2_O_2_. (**f**) CLSM-based imaging of NIH-3T3 cells under different experimental conditions to assess ROS levels. (**g**,**h**) Viability assays of NIH-3T3 cells following exposure to FePS_3_ NSs at various concentrations, under oxidative stress induced by Rosup (**g**) or H_2_O_2_ (**h**). (**i**,**j**) ELISA measurements showing the secretion levels of pro-inflammatory cytokines TNF-α (**i**) and IL-6 (**j**) in RAW264.7 macrophages treated with FePS_3_ NSs at different concentrations, following stimulation with LTA [105]. Reproduced with permission from ref. [105], Copyright 2021, ROYAL SOCIETY OF CHEMISTRY.

Li et al. [105] introduced FePS_3_ NSs as innovative therapeutic nanoagents capable of responding to specific microenvironments associated with bacterial biofilm infections. These NSs exhibit dual functionality: enhanced Fenton reactivity for effective biofilm disruption and ROS scavenging for mitigating inflammation. The NSs were synthesized from bulk FePS_3_ through a two-step process involving ball milling followed by US exfoliation (Figure 5a). FePS_3_ NSs displayed pH-responsive behavior, undergoing dissociation under acidic conditions to release Fe^2+^ and [P_2_S_6_]^4−^, while maintaining structural integrity in neutral environments. Within acidic biofilm-infected tissues, the liberated Fe^2+^ participated in Fenton-type reactions, converting endogenous H_2_O_2_ into highly reactive •OH. Simultaneously, the [P_2_S_6_]^4−^ facilitated the reduction of Fe^3+^ back to Fe^2+^, promoting efficient iron redox cycling and enhancing overall catalytic activity. Conversely, in neutral pH conditions typical of healthy tissues, FePS_3_ NSs exhibited antioxidative behavior, scavenging H_2_O_2_ and •OH through redox interactions mediated by [P_2_S_6_]^4−^. These microenvironment-responsive properties enabled FePS_3_ NSs to perform dual functions, disrupting biofilms through ROS-mediated antibacterial action while simultaneously mitigating inflammation in surrounding healthy tissues, demonstrating their potential as selective and multifunctional therapeutic agents for bacterial biofilm-associated infections (Figure 5b). Quantitative evaluation revealed that treatment with FePS_3_ NSs (50 μg/mL) in combination with H_2_O_2_ (100 μM) reduced the biofilm biomass to approximately 24%, a significantly greater reduction compared to treatments with H_2_O_2_ alone (~95% remaining) or FePS_3_ NSs alone (~50% remaining). This synergistic effect highlights the improved biofilm-disrupting efficiency of the combined system.

Morphological results through SEM further validated these findings, signifying extensive structural damage to biofilms upon dual treatment, in contrast to the relatively normal biofilm structure observed with single treatments (Figure 5c). Untreated biofilms of *S. aureus* displayed a typical spherical morphology with plane, intact cell walls. However, when exposed to FePS_3_ NSs combined with H_2_O_2_, the bacterial surfaces appeared deformed and wrinkled, indicating structural disruption. The Fenton-boosted catalytic activity of FePS_3_ NSs under acidic conditions was further evaluated for its antibacterial efficacy against both free-floating and biofilm-embedded *S. aureus*. Growth curve analysis confirmed a marked suppression of bacterial proliferation following FePS_3_ NSs treatment, both in the presence and absence of H_2_O_2_. To monitor ROS generation within biofilms, the fluorescent probe DCFH-DA was employed. Results from 3D confocal laser scanning microscopy (CLSM) showed a notably stronger fluorescence signal in the group treated with FePS_3_ NSs and H_2_O_2_, compared to treatments with either agent alone, highlighting the superior ROS generation and oxidative stress induced by the synergistic action (Figure 5d). This elevated fluorescence confirms the production of •OH generated through the Fenton activity of FePS_3_ NSs. Calcein acetoxymethyl ester (calcein-AM) staining was employed to visualize live *S. aureus* cells to further evaluate their bactericidal effect within biofilms. Notably, the group treated with FePS_3_ NSs in combination with H_2_O_2_ exhibited the weakest fluorescence signal, indicating a significant reduction in viable bacteria. This result underscores the enhanced antibacterial performance of FePS_3_ NSs under Fenton-reactive conditions within biofilms. Quantitative analysis revealed that treatment with H_2_O_2_ alone led to a modest bacterial inactivation efficiency of just 0.13 log (~25.89%). In contrast, a lower dose of FePS_3_ NSs (27 μM, 5 μg mL^−1^) achieved an enhanced reduction of 0.28 log (~47.64%), highlighting their superior anti-biofilm potential. When biofilms were treated with a higher concentration of FePS_3_ NSs (50 μg mL^−1^) combined with H_2_O_2_ (100 μM), the colony-forming units (CFUs) dropped by approximately 4 logs (~99.99%). In comparison, FePS_3_ NSs alone achieved a reduction of just under 2 logs (~98.62%). These findings demonstrated the robust bactericidal capacity of •OH generated via the Fenton reaction catalyzed by FePS_3_ NSs, particularly in acidic biofilm environments (Figure 5e).

To assess the ROS-neutralizing potential of FePS_3_ NSs in vitro, NIH-3T3 cells were first exposed to Rosup to artificially increase intracellular ROS levels, followed by treatment with FePS_3_ NSs. A marked decrease in fluorescence intensity, measured using DCFH-DA staining, was observed after the addition of FePS_3_ NSs, indicating a reduction in intracellular ROS (Figure 5f). When oxidative stress was induced by Rosup, cell viability dropped to 55% (Figure 5g). However, cells that were subsequently treated with FePS_3_NSs maintained a viability of approximately 95%, reflecting the capacity of NSs to protect cells against ROS-induced damage. Similarly, when H_2_O_2_ was used to induce oxidative injury in NIH-3T3 cells, pre-exposure to FePS_3_ NSs provided notable protection, as reflected in Figure 5h. These findings collectively highlighted the antioxidant role of FePS_3_ NSs in mitigating oxidative cellular damage. Since ROS also functions as a signalling intermediate in inflammation pathways, the anti-inflammatory effect of FePS_3_ NSs, attributed to their ROS-scavenging ability, was further evaluated. In this regard, *S. aureus* lipoteichoic acid (LTA) was used to induce an inflammatory response in RAW264.7 murine macrophages. The effects of the treatment were evaluated by determining the concentrations of two pro-inflammatory cytokines, TNF-α and IL-6. Pretreatment with FePS_3_ NSs substantially lowered both TNF-α and IL-6 levels (Figure 5i,j), suggesting that these nanomaterials can attenuate inflammatory signaling. Thus, FePS_3_ NSs show promise in counteracting both oxidative stress and inflammation under physiological conditions. In summary, this study established FePS_3_ NSs as a distinctive pH-sensitive therapeutic agent that integrates Fe-based catalytic activity and anion-driven ROS regulation, offering a dual-function approach for selective anti-biofilm and anti-inflammatory treatment in bacterial biofilm infections. The current evidence suggests that CDT can provide an effective antimicrobial strategy by exploiting catalytic ROS generation within the local infection microenvironment. However, the majority of reported studies remain at the in vitro or preclinical stage, and further in vivo investigation is required to establish its efficacy, safety, stability, and performance under the variable pH and peroxide conditions encountered in oral tissues before clinical translation can be considered.

The activity of CDT systems is closely dependent on the availability of substrates required for catalytic ROS generation, particularly H_2_O_2_ in peroxidase-like or Fenton-type reactions. However, the source and local concentration of H_2_O_2_ should be considered carefully. Experimental studies may introduce exogenous H_2_O_2_ at concentrations that are not representative of the physiological or pathological oral environment, whereas endogenous H_2_O_2_ may arise from microbial metabolism, host processes, or other biochemical reactions. Some advanced systems have also been designed to generate H_2_O_2_ enzymatically or through catalytic reactions within the infection site. These mechanisms should be distinguished when interpreting CDT efficacy because the availability, concentration, and spatial distribution of H_2_O_2_ can substantially affect catalytic ROS production. In particular, strong antimicrobial activity obtained only after exogenous H_2_O_2_ administration represents an important translational limitation and should not be interpreted as evidence of clinical efficacy without validation under physiologically relevant conditions.

### 3.5. Nanozyme-Based Antibacterial Therapy

Nanozymes, a class of nanomaterials that mimic natural enzymatic activity, have emerged as versatile agents in biomedical research. By combining the efficiency of biological enzymes with the robustness of synthetic catalysts, nanozymes offer a cost-effective, stable, and scalable alternative for diverse medical applications [106]. Their incorporation into dental materials and therapeutic strategies for oral diseases has become significant due to their catalytic efficiency, biocompatibility, and long-term durability. These properties position nanozymes as promising agents for next-generation dental therapies [107,108]. Studies have demonstrated that nanozyme activity can be finely tuned by environmental factors such as pH, metal ion oxidation states, H_2_O_2_ concentration, and GSH levels. This tunability enables their selective application in various biological contexts, including cancer diagnostics, immune system evaluation, and biosensing technologies [109]. Nanozymes exhibit distinct catalytic behaviors that allow for targeted intervention in various clinical manifestations of chronic oral infections. Nanozymes with oxidase (OXD)-like activity and peroxidase (POD)-like activity facilitate the conversion of O_2_ or H_2_O_2_ to ROS that possess cytotoxic properties [110]. Peroxidase-like nanozymes generally catalyze H_2_O_2_-dependent oxidation reactions and can promote the formation of highly reactive species, whereas oxidase-like systems may use molecular oxygen as an electron acceptor and generate reactive oxygen intermediates without requiring externally supplied H_2_O_2_. In contrast, catalase-like and superoxide dismutase-like activities generally contribute to ROS decomposition or antioxidant defense. Their catalytic performance is influenced by substrate availability, pH, temperature, surface chemistry, catalytic-site accessibility, and the local oral microenvironment [111]. Additionally, nanozymes exhibiting superoxide dismutase (SOD)-like activity convert •O^2−^ into H_2_O_2_ and O_2_, thereby reducing excessive ROS levels, mitigating oxidative stress, and alleviating inflammation associated with infections [112]. Finally, nanozymes with catalase-like activity decompose local H_2_O_2_ into O_2_, helping to relieve hypoxic conditions typical of anaerobic infections. In cancer therapy, these nanozymes similarly alleviate tumor-associated hypoxia, thereby enhancing the efficacy of treatment strategies [113,114]. Given the significant involvement of ROS and oxidative stress in the onset and progression of oral diseases, it is essential to conduct in-depth investigations into how nanozymes and associated dental materials function in managing these conditions, focusing on both their therapeutic mechanisms and application strategies.

Liu et al. [115] developed copper-doped carbon dots (Cu-CDs) synthesised under controlled thermal conditions, offering multifunctional applications, including antibacterial activity, biofilm disruption, wound healing, and tooth whitening, for managing oral infectious diseases. These ultrasmall Cu-CDs demonstrated excellent catalase-like and POD-like enzymatic action, facilitating enhanced ROS and O_2_ generation in the oral microenvironment, thereby supporting a wide range of therapeutic actions. An important feature of these nanoenzymes is their strong affinity for lipopolysaccharide (LPS) and peptidoglycan (PGN), which enables effective bacterial capture and localised delivery. This binding proficiency translated into prominent antimicrobial effects against both *S. aureus* and *E. coli*. Moreover, their therapeutic efficiency was corroborated in a rat model for infectious wound healing. Cu-CDs also effectively cleared *S. mutans* biofilms from tooth surfaces, combining ROS-mediated chemical action with O_2_ bubble-induced mechanical disruption.

**Figure 6:**
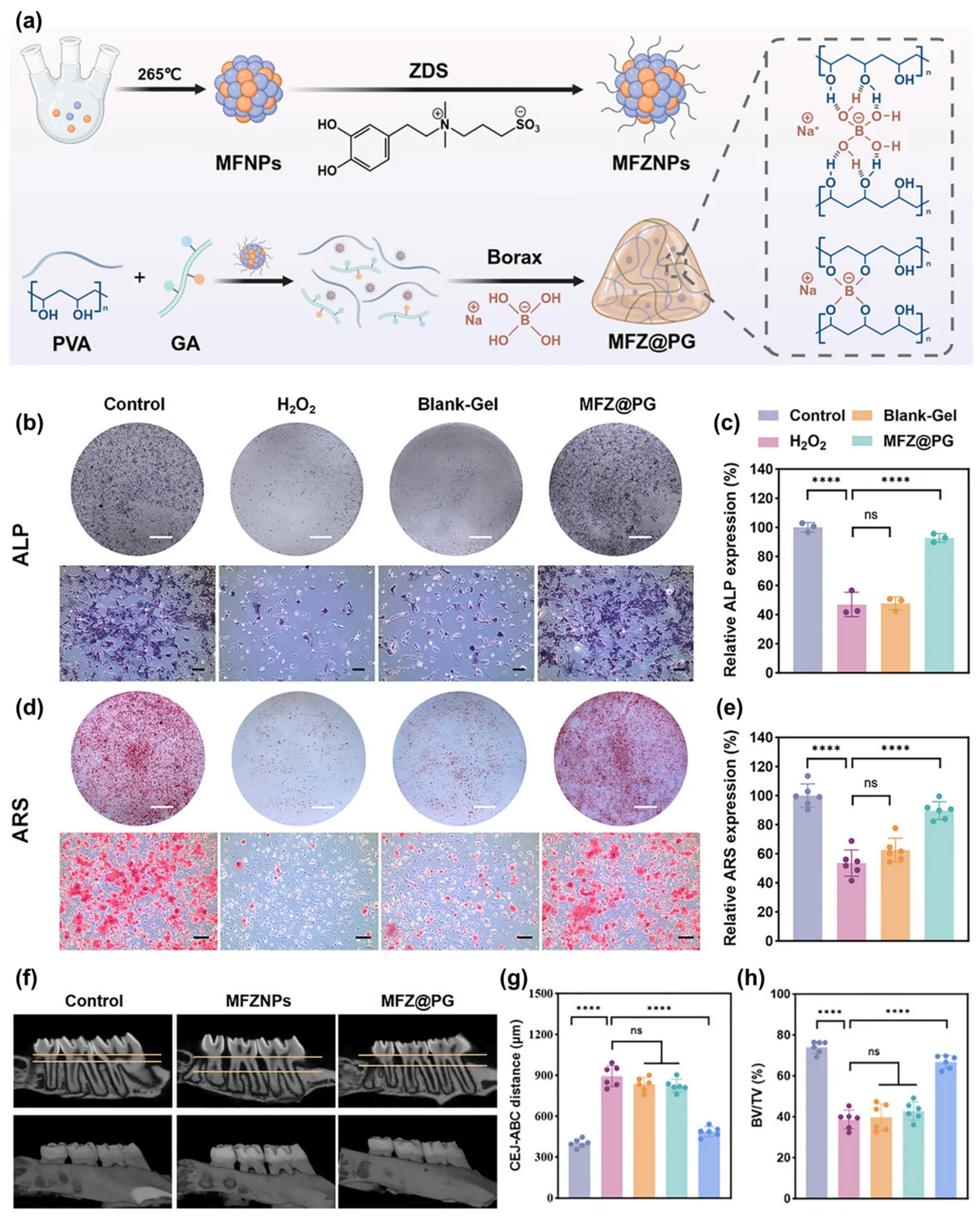
(**a**) Schematic representation of the synthesis of ligand-modulated MFZ@PG. (**b**,**c**) Characteristic images and standardized analysis of ALP staining on day 7. Scale bar: white-5 mm, black-200 µm. n = 3 independent replicates. (**d**,**e**) Characteristic images and standardized analysis of ARS staining on day 30. Scale bar: white-5 mm, black-200 µm. n = 6 independent replicates. (**f**) 2D and 3D CT images of the maxillary alveolar bone of the fourth week after MFZ@PG treatment. Assessment of (**g**) CEJ-ABC distance, (**h**) BV/TV ratio. Data are presented as mean ± SD. One-way ANOVA followed by Bonferroni post hoc analysis was used to compare multiple groups. ns, no significant difference, **** *p* < 0.000 [116]. Reproduced with permission from ref. [116], Copyright 2026, WILEY.

Zhang et al. [116] engineered a ligand-regulated manganese ferrite nanozyme hydrogel (MFZ@PG) to address oxidative stress and periodontal tissue destruction through activation of the ZBP1/β-catenin signalling pathway. The hydrogel was fabricated by dispersing manganese ferrite nanozymes (MFZNPs), known for their ROS-scavenging activity, within a polyvinyl alcohol (PVA) and gelatin (GA) network, followed by borax-mediated crosslinking (Figure 6a). Owing to the intrinsic catalytic properties of manganese ferrite and the incorporation of catechol functionalities, MFZ@PG exhibited strong antioxidant activity, while the dynamic interactions among borax, PVA, and gelatin endowed the hydrogel with excellent tissue adhesion. Given the importance of alveolar bone regeneration in periodontitis therapy, the osteogenic potential of MFZ@PG was investigated using MC3T3-E1 cells. Early-stage osteogenic differentiation was assessed by alkaline phosphatase (ALP) staining, whereas late-stage mineralization was evaluated through alizarin red S (ARS) staining. Exposure to H_2_O_2_ markedly suppressed osteogenic activity, reducing relative ALP expression to 46.9% of the control level. Treatment with MFZ@PG largely restored this response, increasing ALP expression to 92.7% (Figure 6b,c). A similar trend was observed for mineralization, where ARS expression increased from 53.6% in the H_2_O_2_-treated group to 89.7% following MFZ@PG treatment (Figure 6d,e), indicating effective protection against oxidative stress-induced impairment of osteogenesis.

The therapeutic efficacy of MFZ@PG was further validated in a ligature-induced rat model of periodontitis. Micro-CT analysis demonstrated substantial alveolar bone regeneration in animals treated with the hydrogel compared with the untreated periodontitis group (Figure 6f). Quantitative assessment showed that the CEJ-ABC distance increased from 402.1 μm in healthy controls to 894.4 μm following disease induction, reflecting severe bone loss. Remarkably, MFZ@PG treatment reduced this value to 487.5 μm, indicating significant recovery of periodontal bone tissue (Figure 6g). Consistently, the BV/TV, a key indicator of bone density, increased from 38.5% in the periodontitis group to 66.8% after hydrogel administration (Figure 6h). Collectively, these findings demonstrate that MFZ@PG effectively alleviates oxidative stress, promotes osteogenic differentiation, and enhances alveolar bone regeneration, highlighting its potential as a clinically translatable therapeutic platform for periodontitis management.

Before clinical translation, the safety of certain nanozymes, especially metal-containing nanozymes, should be evaluated through a stepwise framework that considers both the intact nanomaterial and its degradation products. In addition to conventional cytotoxicity assays, studies should assess material dissolution, metal-ion release, biodegradation kinetics, biodistribution, organ accumulation, local inflammatory responses, and short- and long-term toxicity. Particular attention should be given to systemic exposure following local dental administration and to the mechanisms through which the material or its degradation products are cleared from the body. Standardized testing across relevant oral cell models and appropriate animal systems will be essential to establish a reliable safety profile. Progress toward clinical use will depend on rigorous in vivo validation, standardized safety evaluation, reproducible manufacturing, and ultimately well-controlled clinical studies.

Antimicrobial therapeutic strategies targeting dental infections represent a significant advancement beyond conventional antibiotic use. Each of these modalities employs a distinct mechanism of action, ranging from localized hyperthermia in PTT, ROS-mediated killing in CDT, PDT, and SDT, to nanozymes mimicking enzymatic activity to kill bacteria and disrupt biofilms. Despite their potential, challenges such as light penetration depth, potential tissue overheating, exogenous stimulation, and long-term biocompatibility of catalytic nanomaterials must be carefully addressed. Collectively, these strategies reflect the ongoing shift toward precise antibacterial interventions that reduce the development of resistance, yet their clinical acceptance will depend on balancing efficiency with safety and cost-effectiveness. Table 1 provides a summary of the therapeutic significance and clinical potential of all these modalities.

Despite the promising antimicrobial activity reported for PDT, PTT, CDT, SDT, and nanozyme-based therapy, their effectiveness should be interpreted carefully because most studies have been conducted using planktonic bacteria or simplified single-species biofilm models. These experimental systems do not fully reproduce the complex environment of mature oral biofilms, which contain diverse microbial communities embedded within an extracellular matrix and exhibit spatial variations in oxygen availability, nutrient concentration, metabolic activity, and antimicrobial susceptibility. Such heterogeneity can influence nanomaterial penetration, ROS generation, catalytic activity, and the accessibility of therapeutic agents to microorganisms located within deeper biofilm regions. Therefore, results obtained from planktonic or simplified biofilm models may overestimate the efficacy of nanotherapeutics under clinical conditions. Future investigations should place greater emphasis on mature multispecies oral biofilms to provide a more clinically relevant assessment of therapeutic performance and to better establish the translational potential of these emerging antimicrobial strategies.

Moreover, PDT, PTT, SDT, CDT, and nanozyme-based therapies are often described as resistance-independent because their antimicrobial effects generally involve multiple physicochemical mechanisms rather than inhibition of a single microbial target. Nevertheless, this does not mean that microorganisms cannot develop adaptive tolerance following repeated or sublethal exposure. Current evidence supporting the long-term absence of adaptive responses remains limited, particularly for clinically relevant repeated-treatment conditions. Future studies should therefore evaluate changes in microbial susceptibility following serial exposure, including potential alterations in oxidative-stress responses, membrane composition, biofilm formation, metabolic adaptation, and community composition. Such studies are necessary before these approaches can be definitively described as having a low propensity for resistance development.

Also, the therapeutic performance of SDT, PTT, and CDT is strongly dependent on the local physicochemical environment and treatment parameters. For SDT, ultrasound frequency, intensity, exposure duration, and cavitation behavior can substantially influence ROS generation and treatment depth. Similarly, the efficacy and safety of PTT depend on the achieved temperature, heating rate, exposure duration, and spatial distribution of heat within the infected tissue. CDT is particularly sensitive to local pH and the availability of hydrogen peroxide or other substrates required for catalytic ROS generation. Oxygen availability may additionally influence ROS-mediated responses and should be considered when comparing different nanotherapeutic systems. Therefore, differences in experimental parameters and local microenvironment should be carefully considered when comparing antimicrobial efficacy across studies, as apparently similar nanoplatforms may produce substantially different outcomes under different treatment conditions.

### 3.6. Combinatory Therapies

Multimodal therapies have emerged as a powerful stratagem in modern medicine, offering substantial advantages over single-modality treatments. This combination of therapies can address various aspects of the disease, providing a more holistic approach. For example, one therapy might target disease-causing pathogens, while another boosts the body’s immune response or reduces the side effects of the treatment. This multifaceted tactic can provide combined effects that are better than the individual therapies. Moreover, combination therapies can minimize the possibility of resistance development, a frequent challenge in treating chronic diseases. Overall, the effectiveness of combinatory therapies relies on their ability to tackle the complexity of diseases from multiple perspectives, leading to a more sustained and effective therapy response. A few of the combinatory therapies for enhanced therapy against dental infection are described below.

#### 3.6.1. Combinatory PTT and PDT

Currently, visible-mediated phototherapy has gained significant attention as a viable approach for combating bacterial biofilm infections, owing to its advantageous features, including spatiotemporal controllability, minimal invasiveness, moderate penetration depth, and evasion of resistance mechanisms [117]. In PTT, localized temperature exceeding 50 °C, generated via visible light irradiation on photothermal conversion materials, can induce bacterial death through hyperthermia-induced protein denaturation, irreversible bacterial destruction, disruption of bacterial membranes, and increased permeability, ultimately leading to effective bacterial eradication [118]. However, conventional PTT approaches have limitations in terms of precise temperature control and lack an internal self-regulatory mechanism, which increases the risk of thermal damage to adjacent healthy tissues or organs connected to the biofilms [119]. PDT, another alternative phototherapy modality, eliminates biofilms by generating ROS under specific wavelength light irradiation with photosensitizers. The hypoxic microenvironment at the biofilms severely limits the efficacy of PDT [120].

In addition, compared to PTT, although the actuating scope is very limited, PDT offers precise and effective treatment with minimal side effects, attributed to the short diffusion distance (<0.1 μm) and limited lifespan of ROS (∼ns) [121]. Furthermore, phototherapeutic agents encounter several practical challenges, including poor water solubility, aggregation-induced quenching effects, off-target interactions, and insufficient selectivity, which collectively result in suboptimal phototherapeutic efficacy [122,123]. Although PTT and PDT have inherent limitations, both are complementary and promising phototherapies. These limitations are noted as the constraints due to the hypoxic microenvironment are prevalent in biofilms faced by PDT, while the challenges in achieving precise heat confinement and the associated risk of off-target damage for PTT are noted [124]. Therefore, a drug delivery system that is adaptable to both PTT and PDT and can switch from PTT to PDT is highly in demand to combat bacterial biofilm infections, which holds the potential to provide a viable path to complete eradication of bacterial biofilms while minimizing side effects on healthy tissue.

**Figure 7:**
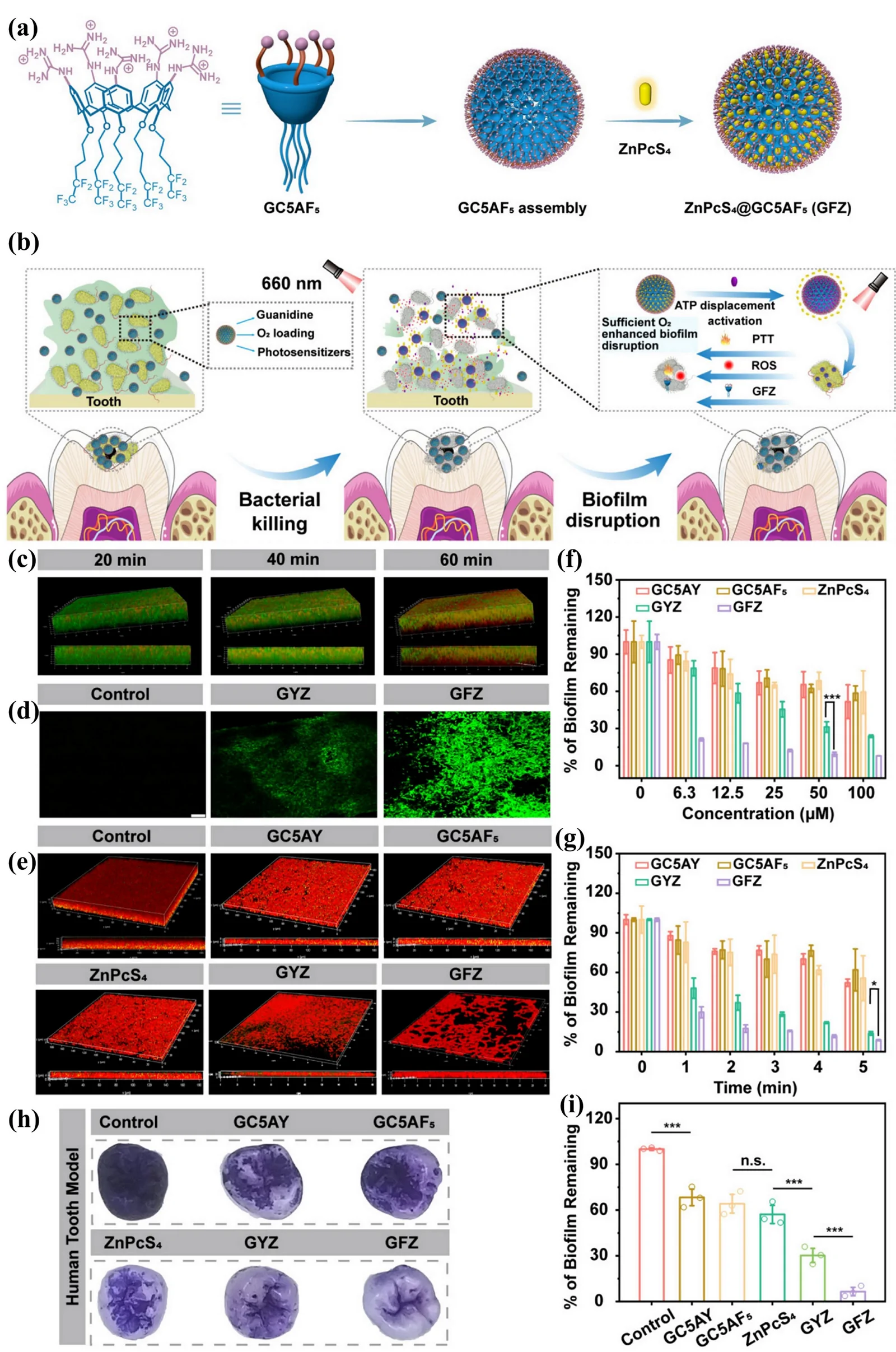
(**a**) Schematic diagram for GFZ preparation. (**b**) Schematic diagram of the ATP-responsive GFZ, designed for enhanced penetration into biofilms and alleviation of biofilm hypoxia, thus boosting oral biofilm dispersal via PTT/PDT. (**c**) CLSM images showcasing the depth-dependent penetration of rhodamine B@GC5AF5 within *S. mutans* biofilms after 20-, 40-, and 60-min treatments. (**d**) Fluorescence intensity of 2′,7′-dichlorodihydrofluorescein serving as a ROS indicator within the biofilm. (**e**) Live/dead fluorescent staining images of the residual biofilms after various treatments. (**f**) Quantitative analysis of the remaining *S. mutans* biofilms across different treatment concentrations. (**g**) Assessment of biofilm reduction under varying durations of 660 nm laser irradiation. (**h**) Effectiveness of *S. mutans* biofilm removal on isolated teeth. (**i**) OD590 values used to evaluate the biofilm eradication efficacy of different sample treatments. *** *p* < 0.001, ns denotes no significance [125]. Reproduced with permission from ref. [125], Copyright 2024, AMERICAN CHEMICAL SOCIETY.

Zhang et al. [125] developed a flexible supramolecular nanoformulation composed of zinc phthalocyanine tetra sulfonate (ZnPcS_4_) and guanidinium-modified calix[5]arene grafted with fluorocarbon chains (GC5AF_5_). The nanoformulation could be switched from PTT to PDT in the presence of adenosine triphosphate (ATP) (Figure 7a). Benefiting from the complexation-induced quenching due to the photoinduced electron transfer effect within the electron-rich cavity of calixarene, preloading of ZnPcS_4_ into the cavity of GC5AF_5_ (referred to as supramolecular nanoformulation ZnPcS_4_@GC5AF_5_, GFZ) resulted in the annihilation of the photodynamic effect (OFF state), while the photothermal effect was enhanced (high state). The activation of the photothermal properties of GFZ through visible irradiation led to bacterial cell membrane rupture and the intracellular ATP release. Afterward, the selective inclusion of ATP stimulated the release of ZnPcS_4_, concurrently restoring the photodynamic activity (ON state) and reducing the photothermal activity (low state). Due to the O_2_-carrying capacity of the fluorocarbon chain in GC5AF_5_, the released ZnPcS_4_, activated upon visible light exposure, generated a significant amount of cytotoxic singlet oxygen (^1^O_2_), thereby accelerating oral bacterial biofilm clearance (Figure 7b).

The primary cause of treatment failure in biofilms is the dense EPS matrix, which severely impedes the delivery of antimicrobial agents into the biofilm structure. To assess the ability of GFZ to penetrate mature biofilms, red fluorescence rhodamine B-loaded GC5AF_5_ was prepared for tracking the delivery of the materials. *S. mutans* biofilms were cultured and incubated with rhodamine B-loaded GC5AF_5_ at 37 °C, and *S. mutans* biofilms were stained with FITC-ConA (green). The results shown in Figure 7c displayed that GC5AF5 completely penetrated the biofilms after 60 min of treatment to reach the enclosed pathogens located deep in the biofilm matrix (≈16 μm). This was consistent with previous studies that guanidine-functionalized nanocarriers quickly penetrate biological membranes due to electrostatic interactions. Improving the hypoxic microenvironment was crucial for increasing the therapeutic efficacy of PDT in bacterial biofilm-associated infections. Given the advantageous oxygen-carrying capacity and superior biofilm penetration of GC5AF_5_, ROS production of biofilms was quantified using 2′,7′-dichlorofluorescin diacetate, transforming into green fluorescent 2′,7′-dichlorofluorescein in the presence of ROS. The intense green fluorescence was observed when *S. mutans* biofilms were treated with GFZ, compared to the GYZ group, under 660 nm light irradiation, suggesting a successfully improved hypoxic microenvironment. (Figure 7d). The results confirm that oxygen transported by GC5AF_5_ significantly enhances the photodynamic effect of ZnPcS_4_, leading to the effective eradication of bacterial biofilms. The fluorescent images of the *S. mutans* biofilms are illustrated in Figure 7e. The control group (PBS) biofilms were thick and exhibited strong green fluorescence. ZnPcS_4_-treated *S. mutans* biofilms under 660 nm light irradiation displayed minimal red fluorescence (EB), indicating limited biofilm disruption.

Notably, the *S. mutans* biofilms after treatment with GYZ under 660 nm light irradiation showed both stronger red and green fluorescence (yellow fluorescence), suggesting an improved biofilm eradication. But the GFZ treatment had the most detrimental effect on the *S. mutans* biofilms, significantly decreasing the number of bacteria and effectively thinning the biofilm. GFZ was able to effectively kill pathogenic bacteria in biofilms under 660 nm light irradiation. The results were also proven via crystal violet staining. Figure 7f showed that compared with the control group, biofilm mass displayed a significant decrease (>90%) after the treatment of 50 μM of GFZ, which was much higher than the biofilm eradication efficiency of the GYZ group (69%). As shown in Figure 7g, GFZ exhibited irradiation-time-dependent antibiofilm effects. Notably, GFZ demonstrated superior dispersion compared to GYZ, achieving an advantageous 65–75% biofilm reduction after just 1 min of 660 nm light exposure. This potent light-driven antibiofilm activity of GFZ likely results from enhanced biofilm penetration by guanidine groups and the ATP-responsive transition from PTT to PDT, effectively eradicating resilient bacterial biofilms.

To further explore the therapeutic potential of GFZ, an ex vivo human biofilm model was employed to evaluate its efficacy in eradicating cariogenic biofilms under 660 nm light irradiation. Therefore, human teeth obtained from the Tianjin Stomatological Hospital were extracted while retaining the crown, infected with *S. mutans* biofilms and treated with nanoformulation and/or 660 nm light for 5 min. The crystal violet staining method was used to observe and quantify remaining biofilms. As revealed in Figure 7h,i, the biofilm dispersion of GC5AF_5_ was about 36%, while those of 660 nm-irradiated GYZ and GFZ were about 70% and 94%, respectively. Overall, the ingenious integration of the recognition, assembly, and O_2_-carrying characteristics of GC5AF_5_ enabled it to be compatible with PTT and PDT, and enabled on-demand switching from PTT to PDT as the treatment progresses, enhancing each treatment mode and more accurately eradicating the bacterial biofilms while avoiding undesired damage to healthy tissues. The supramolecular macrocycles, which help easily achieve functional integration, provide a promising solution for the stubborn bacterial biofilm infections.

#### 3.6.2. Combinatory PTT and CDT

Combining CDT with PTT holds promise as an effective antibacterial therapy for periodontitis. However, this approach has limitations such as inadequate penetration of NIR-I light and complex material structures [126]. Furthermore, the efficiency of existing therapies is limited by the low amounts of endogenous H_2_O_2_ in biofilms and the focus on bacterial eradication rather than colonization prevention. The development of multifunctional synergistic medications, especially those that combine PTT and CDT, has become more prevalent as a means of overcoming these limitations [127]. PTT converts absorbed light energy into localized heat using a photothermal agent. This heat can loosen and disrupt the biofilm structure, facilitating the permeability of •OH. In addition, by raising the local temperature, this combination uses PTT to increase the generation of •OH in CDT, improving therapeutic outcomes [128]. Despite the promise of synergistic CDT and PTT, several limitations hinder their clinical translation. Conventional multifunctional nanomaterials characteristically integrate different components, each with a distinct therapeutic function. This modular structure makes them difficult to fabricate, increases the possibility of cytotoxicity and can affect the trade-off between therapeutic performance and design simplicity. Additionally, current strategies for periodontitis treatment focus mainly on killing bacterial pathogens, often ignoring the role of bacterial co-aggregation in biofilm resistance and disease progression. Furthermore, although H_2_O_2_ is highly expressed at infection sites compared to healthy areas, the endogenous H_2_O_2_ levels alone are insufficient to achieve satisfactory CDT efficiency. This underscores the need for strategies that can enhance H_2_O_2_ levels or improve the utilization of existing H_2_O_2_ to optimize CDT outcomes [129,130].

Lin et al. [131] introduced a multifunctional nanosystem based on Cu_3_P to achieve dual-mode antibacterial therapy by integrating CDT and NIR-II PTT. Cu_3_P nanoparticles were synthesized using a hydrothermal technique and subsequently modified with poly (allylamine hydrochloride) (PAH) to improve aqueous dispersibility and enhance bacterial targeting. To further elevate H_2_O_2_ levels, lactate oxidase (Lox) was incorporated into the construct, leveraging the metabolic byproducts (lactate) of *S. gordonii*. The final nanoplatform, Cu_3_P@PAH@Lox, was designed to harness the synergistic interplay between PTT and CDT. Heat produced by PTT not only damaged bacterial cells directly but also improved the rate of •OH generation via temperature-enhanced Fenton-like reactions, thereby enhancing CDT efficacy. Meanwhile, CDT disrupted bacterial energy supply, the synthesis of ATP and heat shock protein (HSP), increasing bacterial sensitivity to PTT-induced heat. This bidirectional enhancement established an effective feedback mechanism, significantly improving the antibacterial outcome.

**Figure 8:**
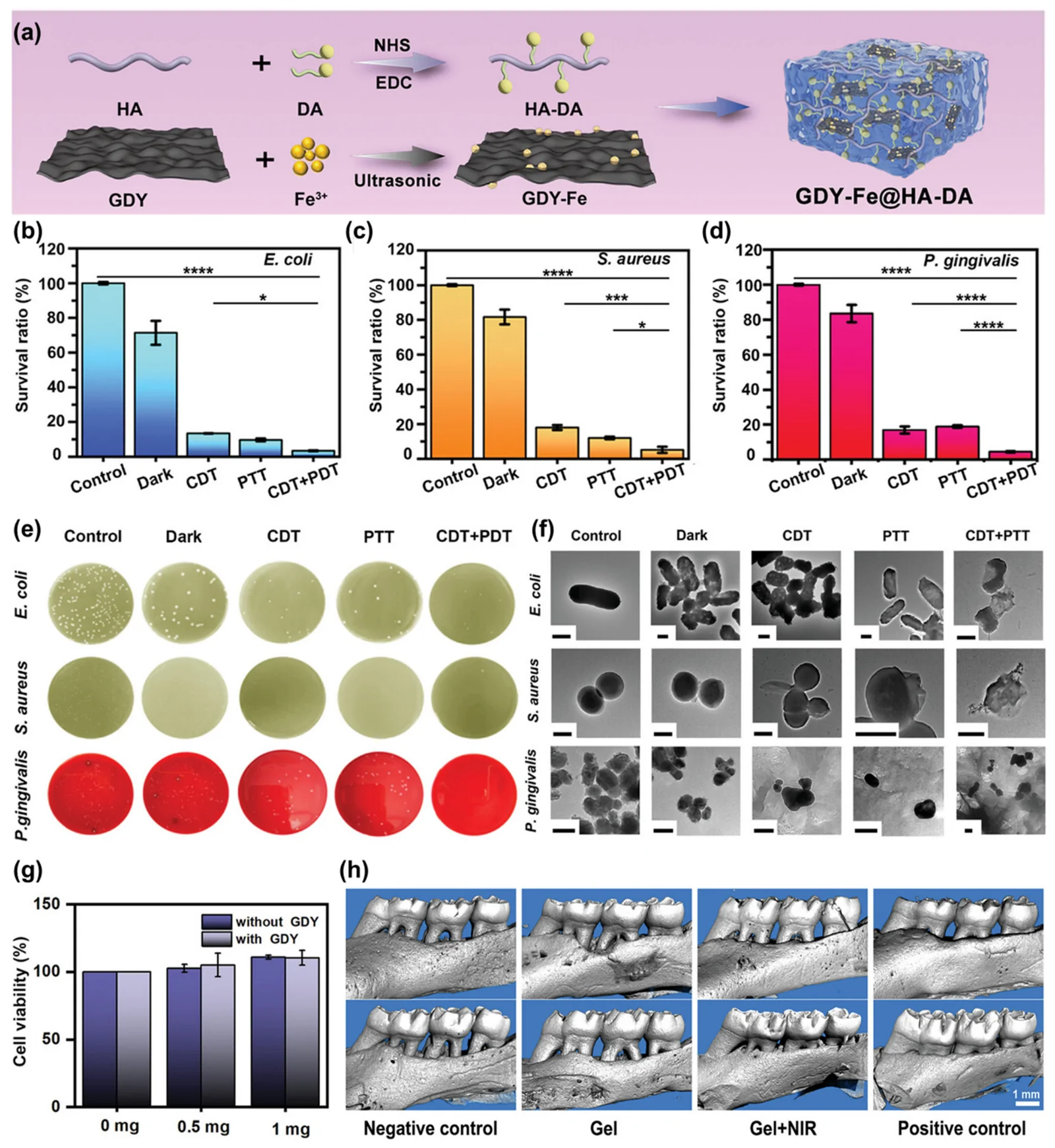
(**a**) Schematic diagram of GDY-Fe@HA-DA hydrogel synthesis. (**b**–**d**) Antibacterial activity of GDY-Fe@HA-DA hydrogel against *E. coli*, S. aureus, and *P. gingivalis* under different treatment conditions. (**e**) Photographs of bacterial plates after different treatments. (**f**) TEM images of *E. coli*, S. aureus, and *P. gingivalis* after different treatments, scale bar: 500 nm. (**g**) Determination of the survival rate of NIH 3T3 cells at 24 h using CCK-8. (**h**) The micro-CT scan of the maxillary molars of rats in different groups after different treatments. (mean ± SD, n = 3, * *p* < 0.05, *** *p* < 0.001, **** *p* < 0.0001, ns denotes no significance) [132]. Reproduced with permission from ref. [132], Copyright 2024, WILEY.

Wu et al. [132] composed an injectable nanoenzyme hydrogel of a dopamine (DA)-modified hyaluronic acid (HA) scaffold and a graphdiyne-iron (GDY-Fe) complex, named GDY-Fe@HA-DA, which exhibited excellent tissue adhesion, self-healing, antibacterial properties, and biocompatibility. Under NIR laser irradiation, GDY-Fe@HA-DA effectively eradicated a variety of pathogens, including *E. coli*, S. aureus, and *P. gingivalis*, through a synergistic combination of CDT and PTT. HA and DA were grafted together to form a natural, non-toxic, and easily degradable HA-DA hydrogel skeleton. Fe^3+^ was loaded on GDY by electrostatic action to obtain GDY-Fe as a nanoenzyme with high Fenton catalytic activity. GDY-Fe@HA-DA hydrogels were then prepared using GDY-Fe as a cross-linking agent for the hydrogel network (Figure 8a). The resulting GDY-Fe@HA-DA hydrogel had the characteristics of being injectable, adhesive, and having controllable mechanical properties, which ensures its convenience and rapidity in the process of use.

The antibacterial properties of GDY-Fe@HA-DA hydrogel were investigated against *E. coli*, S. aureus, and *P. gingivalis* (107 CFU mL^−1^) using the plate counting and live-dead bacterial staining method (Figure 8b–d). Figure 8e displayed the plating medium photos of *E. coli*, S. aureus, and *P. gingivalis* after different processing methods. Compared with the control group, the number of colonies on the culture medium of the dark group decreased slightly, showing an antibacterial efficiency of 15–30%. In contrast, the number of colonies on the media of the single CDT or PTT groups was significantly reduced, and the number of bacterial colonies was reduced by ≈80–90%. However, a single antibacterial method was insufficient to completely kill bacteria, and the antibacterial effect achieved was limited. Therefore, the synergistic effects of CDT and PTT on antibacterial application were studied. The results indicated that the number of colonies on the culture medium of the CDT+PTT group was scarce, especially the colonies of anaerobic *P. gingivalis* were almost invisible, which indicated that the GDY-Fe@HA-DA hydrogel not only had an antibacterial effect on the anaerobic *P. gingivalis* but also played an obvious inhibitory role in the growth of colonies. The bacterial morphology was also characterized after the different treatments using TEM. The surface of the bacterial structure in the control group and the dark group was relatively complete and smooth, without any abnormalities, while the bacteria in the CDT group and PTT group were shrunk, deformed, or even destroyed to different degrees. The bacteria in the CDT+PTT group were wrinkled in morphology, the surface of the cell membrane was seriously damaged, and the contents were leaked (Figure 8f) further demonstrating the excellent antibacterial ability of GDY-Fe@HA-DA hydrogel under the synergistic effect of both CDT and PTT modes. The mouse embryonic fibroblasts (NIH 3T3) as template cells were used to study the biocompatibility of GDY-Fe@HA-DA hydrogel. Cells were cultured in a leachate medium containing GDY-Fe@HA-DA (0, 0.5, and 1.0 mg) hydrogels, and the cell activity was detected at different time points by CCK-8 kit assay. As shown in Figure 8g, adding a small amount of GDY into the hydrogel has no adverse effect on cell proliferation. It even promoted cell proliferation to a certain extent, which showed that GDY-Fe@HA-DA hydrogel has good biocompatibility. SD rats were selected to verify the effect of GDY-Fe@HA-DA hydrogel (NIR) in the treatment of periodontitis in an in vivo experiment. Male SD rats were divided into four groups: negative control group (PBS), Gel group, Gel+NIR group, and positive control group (coated with memantine (Min) + IV vancomycin (Van)). The micro-CT results demonstrated that the GDY-Fe@HA-DA hydrogel exhibited a superior effect in promoting osteogenesis under NIR irradiation in comparison to other treatment groups, which significantly alleviated alveolar bone resorption and bone atrophy near the maxillary second molar (Figure 8h). Overall, this hydrogel can promote cell proliferation to a certain extent, effectively promoting wound healing in mice, reducing inflammatory factors in the periodontal environment of rats, alleviating the periodontal inflammatory environment, and promoting periodontal tissue recovery.

#### 3.6.3. Combinatory PDT and CDT

The combination of CDT and PDT represents a particularly promising strategy for the management of dental infections, especially periodontitis, where dense biofilms, hypoxic microenvironments, and persistent inflammation often limit the effectiveness of conventional treatments. PDT relies on light-activated photosensitizers to generate ROS, whereas CDT exploits endogenous hydrogen peroxide within the infected microenvironment to produce highly cytotoxic •OH through Fenton or Fenton-like reactions. Integrating these two modalities creates a combined therapeutic platform in which CDT continuously amplifies ROS production, while PDT provides spatially controlled antimicrobial activity upon light irradiation. This dual mechanism not only enhances bacterial eradication and biofilm disruption but also overcomes oxygen limitations that frequently compromise PDT efficacy in deep periodontal pockets. Moreover, the elevated oxidative stress generated by the combined treatment can effectively suppress key periodontal pathogens, including Porphyromonas gingivalis, while reducing the risk of antimicrobial resistance associated with traditional antibiotics. Recent nanomaterial-based systems have further strengthened this combination by enabling targeted delivery, sustained catalytic activity, and stimuli-responsive activation within the periodontal microenvironment. Consequently, CDT-PDT combination therapy offers a multifunctional approach that simultaneously addresses microbial burden, biofilm resilience, and disease progression, making it a highly attractive strategy for better periodontal infection management.

**Figure 9:**
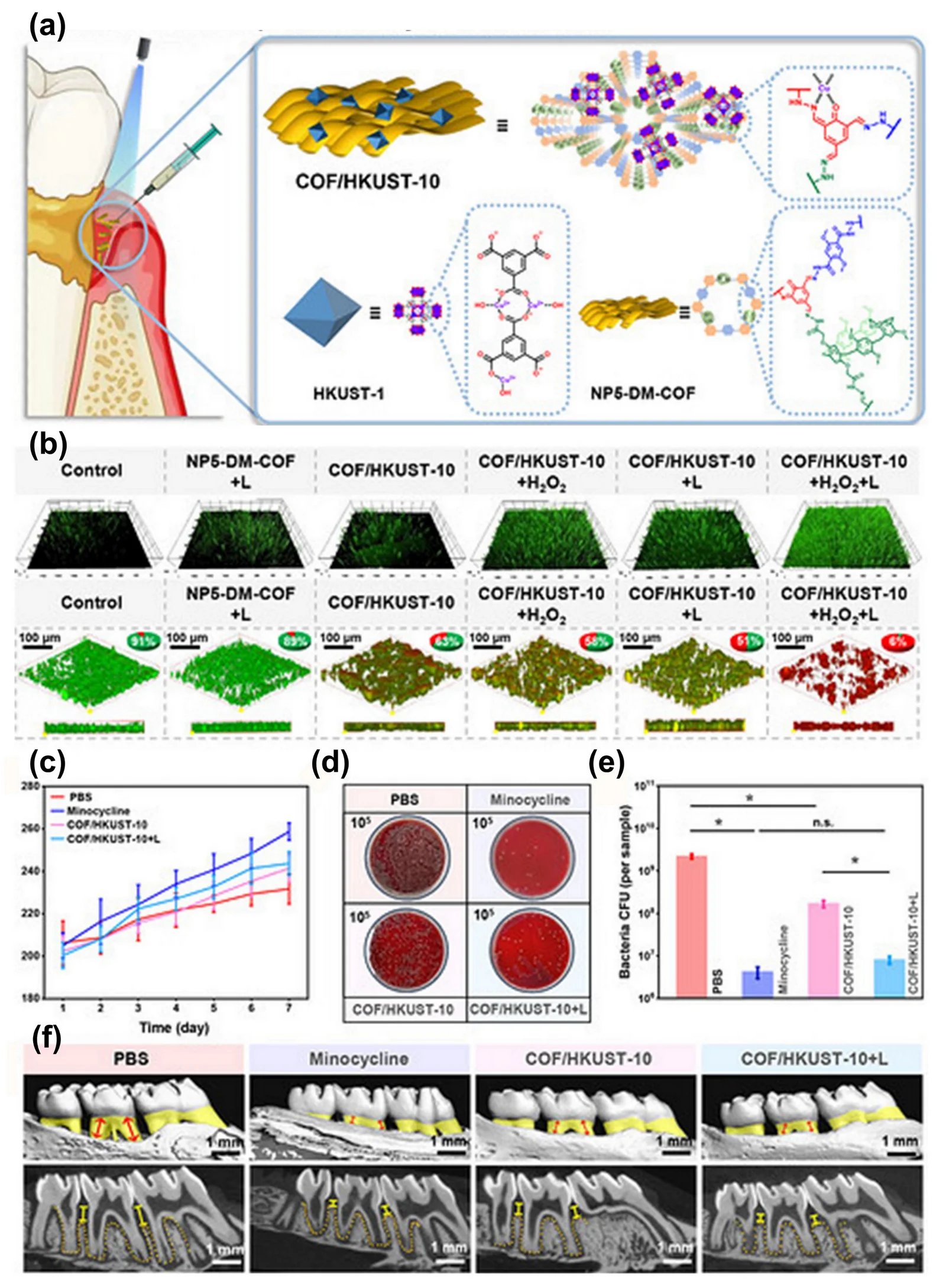
(**a**) Schematic illustration of COF/HKUST-10 fabrication through in situ deposition of HKUST-1 onto NP5-DM-COF. (**b**) DCFH-DA fluorescence imaging of ROS generation within *P. gingivalis* biofilms. (**c**) Body-weight changes of rats during treatment. (**d**) Representative bacterial colonies recovered from the gingival tissues, with dilution factors indicated in the upper left corner. (**e**) Quantitative CFU analysis * *p* < 0.05; n.s.—not significant. (**f**) Micro-CT and 3D reconstructed images showing alveolar bone loss, with red arrows indicating the CEJ-ABC distance [133]. Reproduced with permission from ref. [133], Copyright 2026, WILEY.

Liang et al. [133] developed a multifunctional supramolecular antibacterial platform, termed COF/HKUST-10, to simultaneously deplete H_2_S and amplify oxidative stress for the treatment of periodontitis. The hybrid material was fabricated through the in situ growth of the Cu-based metal-organic framework HKUST-1 within a hydrazone-linked pillararene covalent organic framework (NP5-DM-COF) (Figure 9a). This design enabled a synergistic “three-in-one” antibacterial strategy. The incorporation of conductive HKUST-1 enhanced ROS production through the combined actions of PDT and CDT. Concurrently, depletion of H_2_S reduced the local antioxidant defense capacity of the biofilm microenvironment, thereby intensifying oxidative stress. The generated ROS disrupted bacterial membrane integrity, facilitating intracellular accumulation of Cu^+^/Cu^2+^ ions that interfered with essential metabolic processes and ultimately reduced biofilm viability by approximately four log units. The ability of COF/HKUST-10 to generate ROS within biofilms was investigated using the fluorescent probe DCFH-DA. As shown in Figure 9b, the COF/HKUST-10 + H_2_O_2_ + L group produced the strongest fluorescence signal, followed by the COF/HKUST-10 + H_2_O_2_ and COF/HKUST-10 + L groups, whereas NP5-DM-COF + L and COF/HKUST-10 alone generated substantially weaker responses. These findings confirmed efficient ROS production through multimodal activation under light irradiation.

The in vivo therapeutic performance of COF/HKUST-10 was subsequently evaluated in a ligature- and bacteria-induced rat periodontitis model. Animals were assigned to PBS, minocycline hydrochloride, COF/HKUST-10, and COF/HKUST-10 + L treatment groups and received three administrations on alternate days. Throughout the study, all groups maintained normal body weight gain and feeding behavior (Figure 9c), indicating favorable biocompatibility and negligible systemic toxicity. Subgingival bacterial samples collected after treatment were cultured on Columbia blood agar plates (Figure 9d,e). Consistent with the in vitro observations, the COF/HKUST-10 + L group exhibited a marked reduction in pathogenic bacterial burden compared with the untreated periodontitis group. Notably, its antibacterial efficacy was comparable to that achieved with minocycline, highlighting its therapeutic potential. Micro-CT analysis further demonstrated the ability of COF/HKUST-10 to suppress inflammation-associated alveolar bone destruction. As illustrated in Figure 9f, the PBS-treated group displayed pronounced increases in CEJ-ABC distances at both mesial and distal aspects of the second molar, reflecting severe periodontal bone loss. In contrast, animals treated with COF/HKUST-10 under light irradiation exhibited substantially shorter CEJ-ABC distances and reduced alveolar bone resorption. Collectively, these findings demonstrate that COF/HKUST-10 effectively combines PDT and CDT to eradicate periodontal biofilms, attenuate inflammatory bone loss, and preserve periodontal architecture, providing a promising supramolecular platform for the management of biofilm-associated oral infections.

#### 3.6.4. Combinatory SDT and CDT

Combining SDT with CDT offers a highly effective strategy for treating bacterial dental infections, particularly those involving multidrug-resistant pathogens [134]. SDT uses US waves to activate sonosensitizers, which generate ROS that induce cytotoxicity in bacteria, including those resistant to conventional treatments [135]. This modality is noninvasive and cost-effective, and it provides deeper tissue penetration than phototherapies, making it suitable for targeting deep-seated periodontal pathogens [136]. On the other hand, CDT employs chemical agents that produce ROS or other reactive species to eradicate bacteria. When SDT and CDT are combined, they can significantly enhance antibacterial efficacy by leveraging their synergistic effects [137]. The ROS generated by SDT can potentiate the action of chemodynamic agents, leading to more effective bacterial eradication [138]. This combined approach not only overcomes the limitations of individual therapies, such as low tissue penetration and potential phototoxicity, but also provides a versatile and potent solution for combating complex dental infections, thereby improving clinical outcomes in periodontal disease management [139].

Xin et al. [140] developed a multifunctional nanoplatform that efficiently and noninvasively generates robust ROS against periodontitis by combining CDT and SDT under US irradiation. A dual-layer mesoporous silica nanoparticles (DLMSN) were synthesized using a dual-template technique with deferasirox (DFS) and cetyltrimethylammonium bromide (CTAB) as templates. Subsequently, TiO_2_ was grown in situ on the DLMSNs via a modified hydrothermal process to create mesoporous TiO_2_ (DT). The surface of DT was then coated with Ag by a photocatalytic process to yield the inorganic nanoparticle-based sonosensitizer, DT-Ag. To enhance the antibacterial performance, a positively charged biopolymer chitosan (CS^+^) was coated on the DT-Ag nanoparticles, producing the composite DT-Ag-CS^+^. The highly positive surface charge of CS^+^ significantly enhanced nanoparticle adhesion and facilitated deeper penetration into bacterial cells. Upon US exposure, the DT-Ag-CS^+^ system generated substantial levels of ROS, reflecting potent combined SDT and CDT capabilities. The antibacterial effect of DT-Ag-CS^+^ efficiently prevents the alveolar bone resorption of periodontitis, confirming that DT-Ag-CS^+^, under SDT and CDT, offers a potent synergistic antibacterial and regenerative platform, particularly effective against periodontal pathogens.

**Figure 10:**
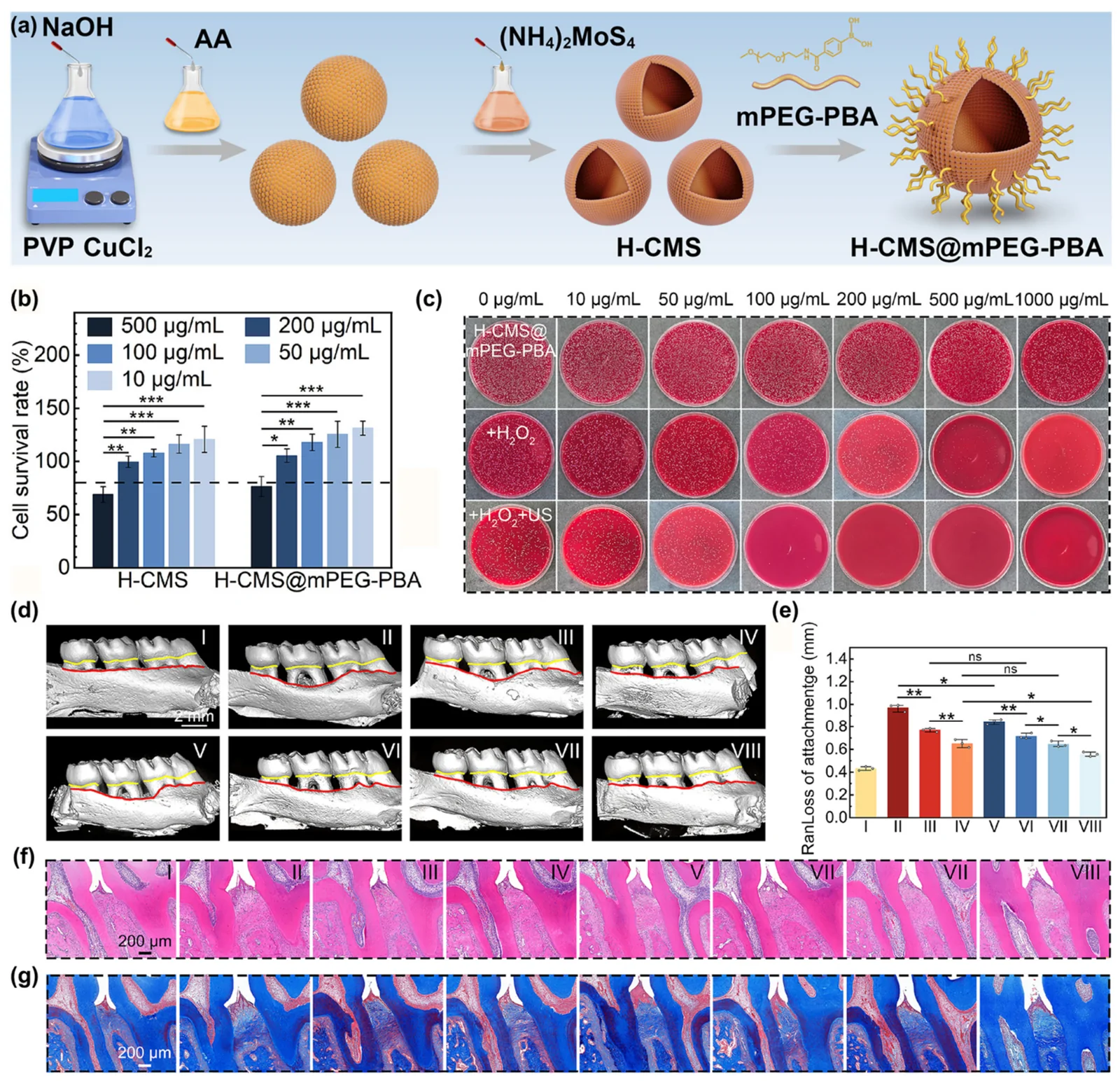
(**a**) Schematic illustration of the synthesis of the multifunctional antibacterial platform H-CMS@mPEG-PBA. (**b**) Cytocompatibility evaluation using L929 cells. (**c**) Antibacterial activity of H-CMS@mPEG-PBA at different concentrations following H_2_O_2_ addition and ultrasound stimulation. (**d**) Micro-CT images of periodontal tissues, where the yellow line indicates the cementoenamel junction and the red line represents the alveolar bone crest. (**e**) Quantitative analysis of attachment loss. (**f**) H&E staining of periodontal tissues. (**g**) Masson staining showing collagen deposition and tissue repair. * *p* < 0.05, ** *p* < 0.01, *** *p* < 0.001, ns denotes no significance [141]. Reproduced with permission from ref. [141], Copyright 2026, ELSEVIER.

Wu et al. [141] designed a copper-molybdenum bimetallic nanoplatform, denoted as H-CMS@mPEG-PBA, for periodontitis treatment through the synergistic integration of SDT and CDT (Figure 10a). The therapeutic concept relies on a dual ROS-amplification mechanism that establishes a self-reinforcing oxidative cycle within the periodontal microenvironment. Initially, ROS generation is driven by the intrinsic oxidase-like (OXD), peroxidase-like (POD), and catalase-like (CAT) catalytic cascade of the nanoplatform. More importantly, US stimulation not only activates SDT but also enhances the OXD-like and POD-like catalytic activities of H-CMS@mPEG-PBA. Meanwhile, oxygen generated through CAT-like reactions further supports sonodynamic ROS production. This reciprocal enhancement between SDT and CDT establishes a positive feedback process capable of generating a localized ROS burst within periodontal pockets. Such a strategy enables efficient bacterial eradication while avoiding excessive oxidative injury to surrounding healthy tissues, thereby improving therapeutic precision and safety. Before biological evaluation, the cytocompatibility of H-CMS and its modified derivative H-CMS@mPEG-PBA was assessed using L929 fibroblasts after 24 h of incubation. As shown in Figure 10b, cell viability remained above 80% when the concentration of either material was below 100 μg mL^−1^, indicating acceptable biocompatibility for subsequent therapeutic applications. The antibacterial performance of H-CMS@mPEG-PBA was then examined under different experimental conditions (Figure 10c). In the absence of external stimulation, the material displayed minimal antibacterial activity. Upon the addition of H_2_O_2_, noticeable bacterial inhibition emerged at concentrations exceeding 500 μg mL^−1^. When ultrasound irradiation was further introduced, antibacterial efficacy increased substantially, with effective bacterial suppression observed at concentrations above 100 μg mL^−1^. This enhanced activity was attributed to the POD-like and CAT-like catalytic processes that continuously converted H_2_O_2_ into highly reactive •OH, while US further amplified ROS production through sonodynamic activation.

The therapeutic potential of the platform was subsequently validated in a rat periodontitis model (Figure 10d). Micro-CT analysis revealed severe alveolar bone destruction in untreated animals, whereas the group receiving the combined H-CMS@mPEG-PBA + H_2_O_2_ + US treatment (Group VIII) exhibited the lowest degree of periodontal attachment loss. Quantitative measurements confirmed that all treatment groups significantly reduced attachment loss relative to the periodontitis control group (0.903 ± 0.012), with Group VIII showing the most pronounced improvement, decreasing attachment loss to 0.546 ± 0.074 (*** *p* < 0.001) (Figure 10e). Histological examination further supported these findings. H&E staining demonstrated reduced epithelial hyperplasia, diminished inflammatory cell infiltration, and improved periodontal tissue architecture following combination therapy (Figure 10f). Consistent with these observations, Masson staining revealed abundant collagen fiber deposition in Group VIII, indicating enhanced repair and remodeling of periodontal tissues (Figure 10g). Collectively, these results demonstrate that H-CMS@mPEG-PBA, when activated by H_2_O_2_ and US, effectively integrates SDT and CDT to achieve potent antibacterial activity, suppress periodontal inflammation, and promote tissue regeneration. The favorable biosafety profile and strong therapeutic efficacy observed in both in vitro and in vivo studies highlight the promise of this platform as a next-generation strategy for periodontitis management.

Although combining complementary mechanisms may improve antimicrobial performance or overcome limitations associated with individual therapies, multifunctional systems can also introduce additional challenges. Increasing the number of active components may complicate synthesis, reproducibility, characterization, sterilization, dose optimization, and large-scale manufacturing. Simultaneous activation of multiple therapeutic mechanisms may also increase the risk of host-tissue injury. Consequently, combination strategies should be evaluated not only according to their antimicrobial benefit but also according to interaction mechanism, safety, formulation complexity, and clinical feasibility.

Moreover, the absence of evident cytotoxicity or inflammatory responses does not necessarily indicate complete tissue compatibility. ROS-generating combination strategies may still cause local tissue irritation through transient oxidative stress, membrane interactions, or prolonged exposure, particularly when ROS generation is insufficiently confined to the infected site. Therefore, future studies should evaluate local irritation and mucosal tolerance together with cytotoxicity and inflammatory responses, including histopathological examination and longer-term tissue recovery in relevant oral models. Establishing controlled ROS generation and an appropriate therapeutic window will be essential to ensure antimicrobial efficacy without compromising surrounding healthy tissues. Overall, these nanotherapeutic strategies show promising potential for dental infection management, but their efficacy and translational readiness vary with the infection model, biofilm complexity, treatment conditions, and safety profile (Table 2).

## 4. Regenerative Strategies for Infection-Induced Dental Tissue Defects

The regeneration of damaged or necrotic tissues resulting from trauma or disease poses a substantial challenge in biomedical science. The dental pulp, a central, highly organized tissue vital for maintaining tooth vitality, can undergo irreversible damage due to caries, periodontal infections, or traumatic injury, regardless of the stage of root development [142]. Notably, dental tissues harbor mesenchymal stem cells with inherent regenerative potential, offering a promising avenue for pulp tissue repair [143,144]. Currently, tooth loss is addressed through prosthetic replacements such as dentures and implants, or bone grafts in cases of alveolar bone resorption [145]. Meanwhile, innovations in adhesive materials, particularly in polymer and ceramic sciences, have improved enamel bond strength and durability. Nevertheless, the challenge of restoring enamel and addressing its degradation persists. Despite stronger adhesion to enamel than dentin, restorative materials still face limitations such as polymerization shrinkage, susceptibility to recurrent caries, and long-term mechanical degradation [146]. Although these treatments provide structural support, they remain symptomatic solutions. In contrast, tooth regeneration is an ideal biological solution to restore the complete structure and function of lost teeth and supporting tissues. Recent developments have increasingly focused on multifunctional regenerative nanobiomaterials capable of addressing both infection and tissue damage within a single platform. Protein- and amyloid-based supramolecular systems can provide tunable self-assembly, cellular interactions, and structural support while incorporating antimicrobial functionality, making them attractive for infected tissue regeneration. Similarly, metallic nanomaterials and nanocomposites can combine broad-spectrum antimicrobial activity with physicochemical properties that support cell adhesion, tissue repair, and wound healing. Such integrated platforms are particularly relevant to dental applications, where effective infection control must be accompanied by restoration of damaged soft or hard tissues. Recent studies have demonstrated that the rational combination of antimicrobial and regenerative functions within supramolecular and nanocomposite architectures can provide a more comprehensive therapeutic response than single-function materials.

**Figure 11:**
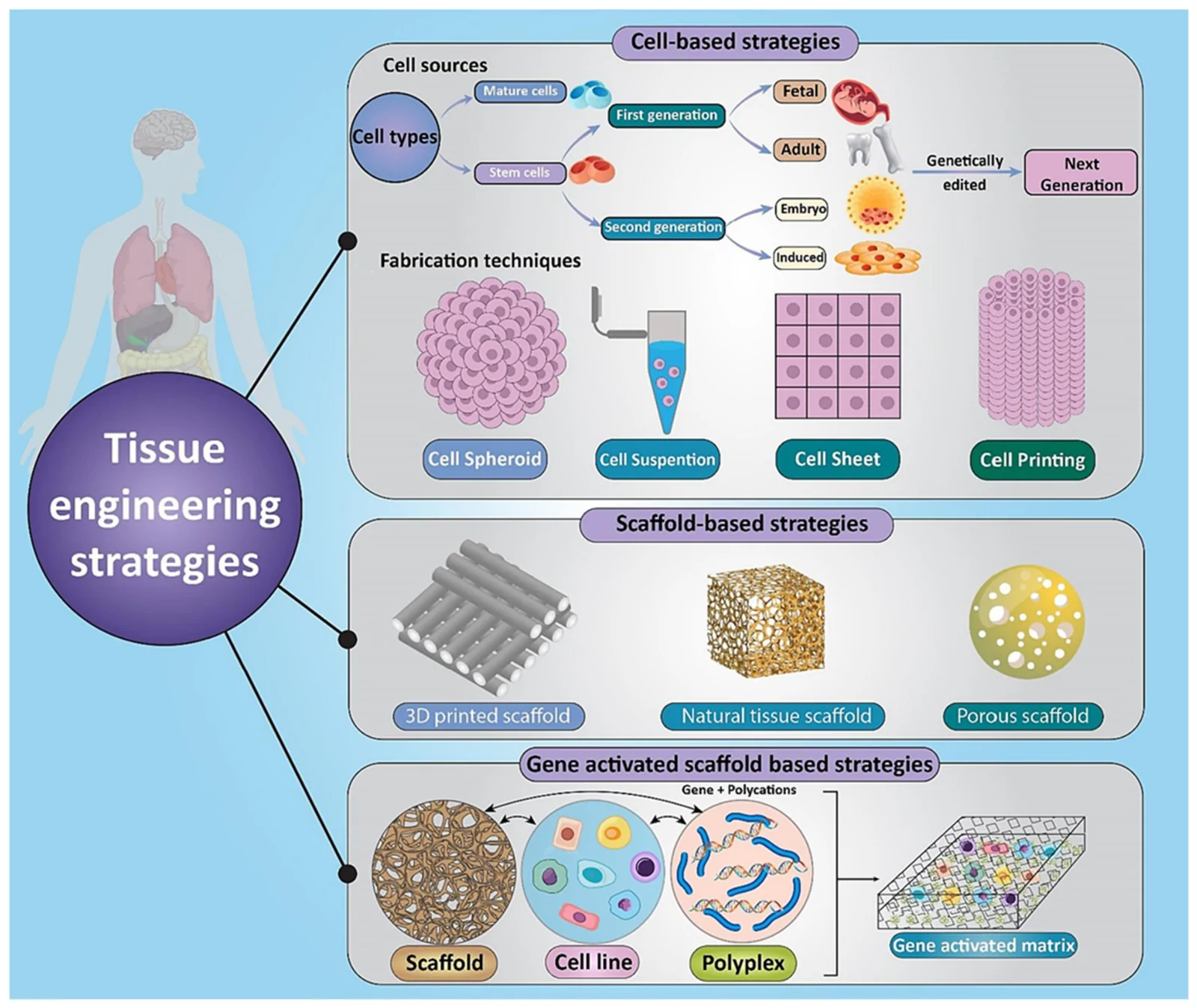
Diagrammatic representation of tissue engineering strategies: gene-activated scaffold-based, scaffold-based, and cell-based [148]. Reproduced with permission from ref. [148], Copyright 2023, ELSEVIER.

Significant progress in tissue engineering over the past few years has enabled its application in dentistry, especially within regenerative endodontics. Successful tissue regeneration depends on the biochemical and topographical characteristics of the cellular microenvironment, ranging in scale from nanometers to micrometers [147]. Advances in biomaterial science, especially smart materials, nanostructures, and 3D-printed scaffolds, have significantly accelerated progress in dental tissue engineering. These scaffolds are designed to replicate the extracellular matrix, facilitating essential cellular behaviors including adhesion, proliferation, and differentiation, thus enhancing tissue integration and regeneration. Scaffolds serve not only as structural templates but also as bioinstructive platforms capable of recapitulating the mechanical and biochemical cues of native tissue. To this end, various strategies have been developed: direct implantation of autologous cells into damaged sites; localized delivery of growth factors or bioactive molecules via scaffold systems; acellular scaffolds that recruit host cells; cell-laden constructs that mimic native extracellular architecture; and scaffold-integrated gene or protein therapies that guide regenerative outcomes (Figure 11) [148]. By combining bioactive molecules and maximizing tissue compatibility and functionality, these panels represent the comprehensive approaches in bioengineering for improved tissue integration and regeneration, which are in line with state-of-the-art biomedical innovations and have the potential to completely transform dental care.

**Figure 12:**
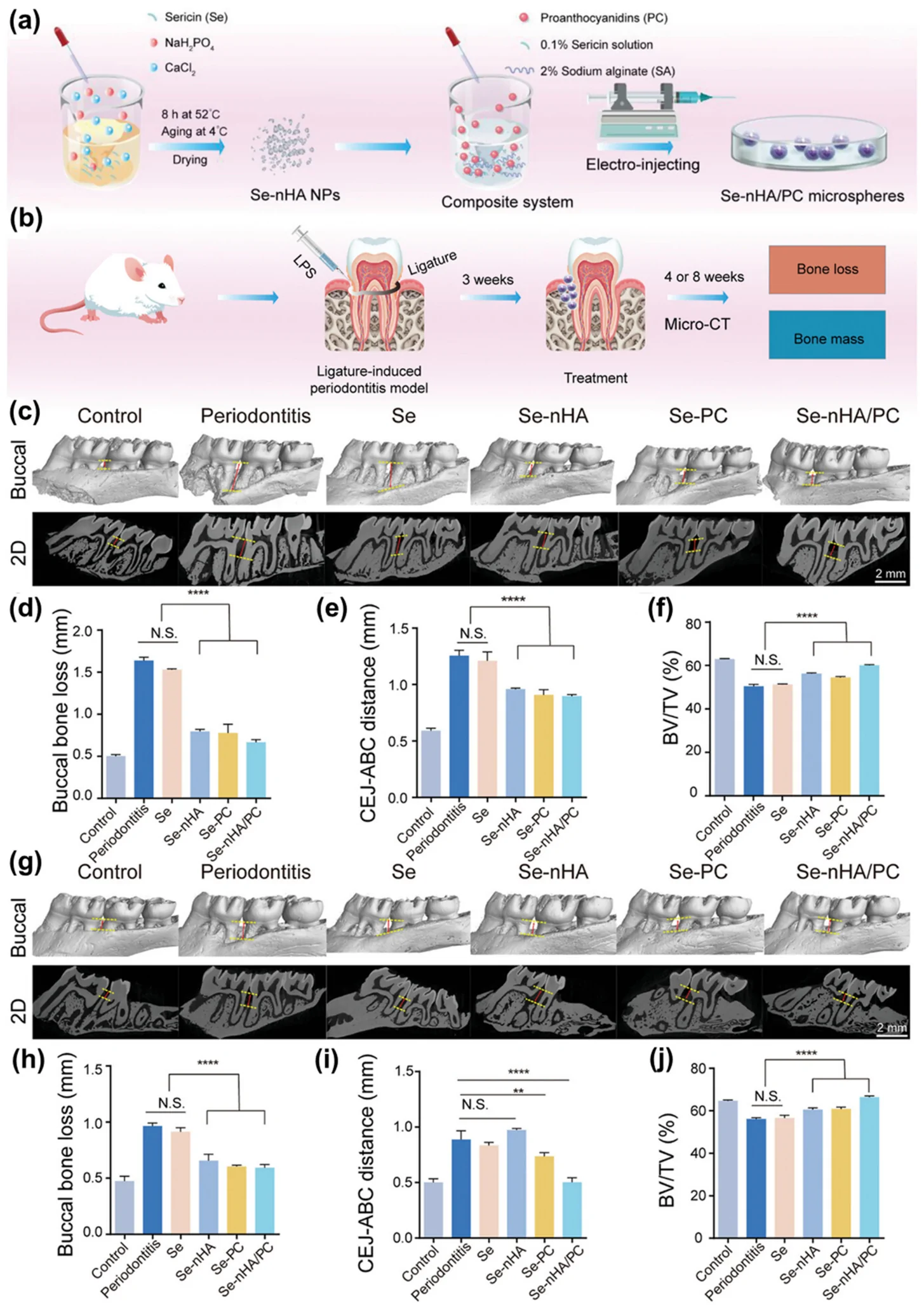
(**a**) Synthesis of Se-nHA NPs and Se-nHA/PC microspheres. Assessments of in vivo bone regeneration. (**b**) Micro-CT experiment flow diagram. (**c**) Four-week post-implantation 2D and 3D reconstruction images (upper dashed line: CEJ, lower dashed line: ABC, red line: bone loss). (**d**) Quantitative estimation of the height of lateral buccal bone loss. (**e**) Distance between CEJ-ABC. (**f**) Micro-CT images calculation for BV/TV. (**g**) Eight-week post-implantation 2D and 3D reconstruction images. (**h**) Quantitative estimation of the height of lateral buccal bone loss. (**i**) Distance between CEJ-ABC. (**j**) Micro-CT images calculation for BV/TV. ** *p* < 0.01, **** *p* < 0.0001, N.S. denotes no significance [153]. Reproduced with permission from ref. [153], Copyright 2025, WILEY.

Therefore, there is a growing demand for innovative strategies that can promote bone regeneration in the context of periodontitis. Drawing inspiration from bone biology, the development of scaffold materials that combine immunomodulatory and osteoinductive capabilities holds promise for addressing alveolar bone loss [151,152]. In a recent study, Ming et al. [153] employed a biomimetic mineralization strategy using sericin as a protein template to synthesize selenium-doped nanohydroxyapatite nanoparticles (Se-nHA NPs). These NPs, along with proanthocyanidins (PC), were incorporated into a sericin/sodium alginate matrix and then crosslinked via electrostatic interaction in a calcium ion solution, resulting in Se-nHA/PC composite microspheres (Figure 12a). In vitro investigations demonstrated that these microspheres exhibit strong ROS-scavenging capacity, antioxidative effects, and anti-inflammatory activity. They also induced the M2 polarization of macrophages and enhanced the osteogenic ability of human periodontal ligament stem cells (hPDLSCs) in the conditioned media of macrophages.

To assess their therapeutic potential in vivo, the Se-nHA/PC microspheres were tested in a rat model of chronic periodontitis. Results showed that the microspheres effectively regulated macrophage polarization and facilitated osteogenesis of hPDLSCs. Significantly, the microspheres also enhanced the restoration of hard and soft tissues in periodontal defects, as demonstrated by microscopic and macroscopic analysis (Figure 12b). 2D and 3D reconstructions of the alveolar bone (Figure 12c–g) were used to quantify tissue regeneration. Bone loss was evaluated by measuring the linear distance between the distal CEJ of the first molar and ABC. A significant gap, larger than the 0.2 mm diameter of the ligature wire, was observed between the molars of the periodontitis group, indicating severe bone resorption and successful induction of periodontitis (Figure 12c,d). CEJ-ABC assessments in the control, periodontitis, Se, Se-nHA, Se-PC, and Se-nHA/PC groups were 0.59 ± 0.03, 1.25 ± 0.06, 1.21 ± 0.07, 0.96 ± 0.01, 0.9 ± 0.03, and 0.89 ± 0.01 mm, respectively, at the 4-week mark (Figure 12e). After 8 weeks, these values had reduced to 0.50 ± 0.03, 0.88 ± 0.07, 0.83 ± 0.03, 0.87 ± 0.02, 0.73 ± 0.04, and 0.50 ± 0.04 mm, respectively (Figure 12i). These results demonstrate that the Se-only group had a minimal effect on tissue regeneration, whereas the groups treated with Se-nHA, Se-PC, and especially Se-nHA/PC microspheres, showed significant recovery of alveolar bone volume. Among them, the Se-nHA/PC group exhibited CEJ-ABC distances most comparable to those of the control, indicating superior bone height restoration. Moreover, the Se-nHA/PC group showed optimal regeneration outcomes, characterized by well-integrated soft tissues and enhanced alveolar space (Figure 12d,h). Improvements in trabecular bone architecture were also evident. The BV/TV ratio in the treated regions was comparable to that of healthy controls, signifying considerable bone density recovery and significant inhibition of bone resorption (Figure 12f,j). Overall, this study highlights the potential of Se-nHA/PC microspheres as a multifunctional biomaterial that can regulate immune modulation and osteogenesis, offering a promising solution for periodontal tissue regeneration in inflammatory microenvironments.

While traditional biomaterials like polymers, ceramics, and natural scaffolds contribute structural and biocompatible support, tooth regeneration still represents one of the most demanding challenges in dental medicine, requiring biomaterials that not only restore lost tissue but also actively guide cellular behavior and immune balance within the complex oral environment [154]. Piezoelectric materials and composites are emerging in this, as these add an active, dynamic dimension in tooth regeneration that addresses impaired cell function and chronic inflammation, which are the two major barriers to successful regeneration. Piezoelectric materials are promising because they can convert mechanical forces from natural activities such as chewing into localized electrical cues that stimulate cellular differentiation, tissue remodelling, and bone or dentin repair. This self-powered capability circumvents the need for external wiring or invasive devices, offering a clinically adaptable approach [155,156,]. Li et al. [157] developed a P(VDF-TrFE) piezoelectric film incorporated with 2 wt% SrCl_2_, which exhibited excellent flexibility, favorable biocompatibility, and a notable piezoelectric d33 coefficient of 14 pC^−1^. These properties generated a localized electric microenvironment capable of attracting dental pulp stem cells (DPSCs) and directing their differentiation into odontoblasts under physiological activities such as chewing and speaking. Meanwhile, the controlled release of Sr ions from the film also increased the odontogenic differentiation of DPSCs.

Notably, piezoelectric platforms also have the ability to modulate immune responses, to convert inflammatory microenvironments into regenerative ones, while also promoting stem cell differentiation into odontoblasts or osteoblasts. This multi-tasking (bioelectrical stimulation and immunodulation) makes piezoelectric platforms very promising for pulp-dentin and periodontal regeneration [158,159,160]. Pan et al. [161] engineered Al-doped strontium titanate/titanium dioxide nanotubes (Al-SrTiO_3_/TiO_2_, Al-STNT) as an ultrasound-responsive coating on titanium implant surfaces. The substitution of Al^3+^ within the SrTiO_3_/TiO_2_ heterojunction created oxygen vacancies and lattice distortions, lowering the bandgap and enabling enhanced piezoelectric activity under ultrasonic stimulation. This process produced significant ROS, which efficiently disrupted bacterial biofilms and inhibited their metabolic activity. Beyond antibacterial function, the nanoscale SrTiO_3_ coating also promoted osteogenic activity, facilitating robust osseointegration between implants and alveolar bone. In a rat model mimicking human dental implants, Al-STNT exhibited substantial osseointegration after implantation, as well as its efficient antibacterial activity as a sonosensitizer. Moving forward, the use of piezoelectric biomaterials in restorative and implant treatments holds great promise for the future of regenerative dentistry and may lead to functional, responsive, and durable solutions for patients.

The regenerative strategies discussed are considered primarily in the context of dental infection management and the restoration of tissues damaged by infection or antimicrobial treatment. Accordingly, the focus is not on dental tissue engineering as an independent field, but on nanomaterials that either combine anti-infective and regenerative functions or support tissue recovery following infection control. Such multifunctional platforms may be particularly valuable in periodontal, pulpal, and peri-implant conditions, where microbial elimination alone may not be sufficient to restore damaged tissue.

## 5. Role of Artificial Intelligence in Dental Healthcare Management

Artificial intelligence (AI) is rapidly advancing the field of dentistry by enhancing diagnostic accuracy, optimizing treatment planning, and modernizing educational practices [162]. Through the application of deep learning algorithms, including machine learning (ML) and image-based data analysis, AI systems have demonstrated strong performance in identifying oral pathologies, supporting clinicians in making more precise and timely decisions [163]. These technologies enable personalized treatment approaches by leveraging large-scale patient data, contributing to improved clinical outcomes and operational efficiency [164]. In dental education, AI is integrated into both didactic and clinical training environments. Virtual simulations, intelligent image interpretation tools, and telemonitoring platforms are equipping students with hands-on experience in diagnostic reasoning and treatment planning [165]. Furthermore, AI streamlines standard administrative procedures such as patient documentation, scheduling, and data management, allowing institutions to employ further resources on direct patient care and academic quality. AI is playing an emerging role in dental research, particularly in data mining, literature synthesis, and manuscript preparation, facilitating faster and more accurate knowledge dissemination [166].

The combination of AI and robotics has led to the creation of fully automated systems capable of synthesizing therapeutic materials without human involvement [167]. The effectiveness of diagnostic and treatment processes is multifaceted, and Fryback and Thornbury have proposed a comprehensive framework for evaluating this effectiveness, which includes technical performance and societal impact [168]. This framework illustrates various levels of deployment in dentistry, each contributing to the understanding of how imaging technologies can improve patient care and inform healthcare policy decisions. The increase in the use of AI in everyday life, including dentistry, is considerable. Hospitals are transitioning from simple information systems to Smart Hospitals and AI hardware and software are rapidly evolving. Ultimately, common AI algorithms like natural language processing (NLP), computer vision (CV), and data mining will be integrated into medical equipment [167].

Liu et al. [169] devised an automated system that uses a deep learning-based object detection technique to detect the marginal bone loss surrounding dental implants. The study complied with institutional ethical requirements and was authorized by the Peking University School and Hospital of Stomatology’s bioethics committee (PKUSSIRB-201837103). To guarantee data quality, the study used 2500 digital periapical radiographs of bone-level implants, with particular inclusion and exclusion criteria. The Faster R-CNN model employed in the study was able to detect bone loss areas similarly to the ground truth bounding box, with performance improving as the severity of bone loss increased. The efficiency of the model was equivalent to that of an amateur dentist, but not as precise as that of an experienced dentist. This study determined that Faster R-CNN could detect peri-implant bone loss and has the potential to assist in the advancement of accurate diagnostic tools, with future developments possible through better-quality training images.

**Figure 13:**
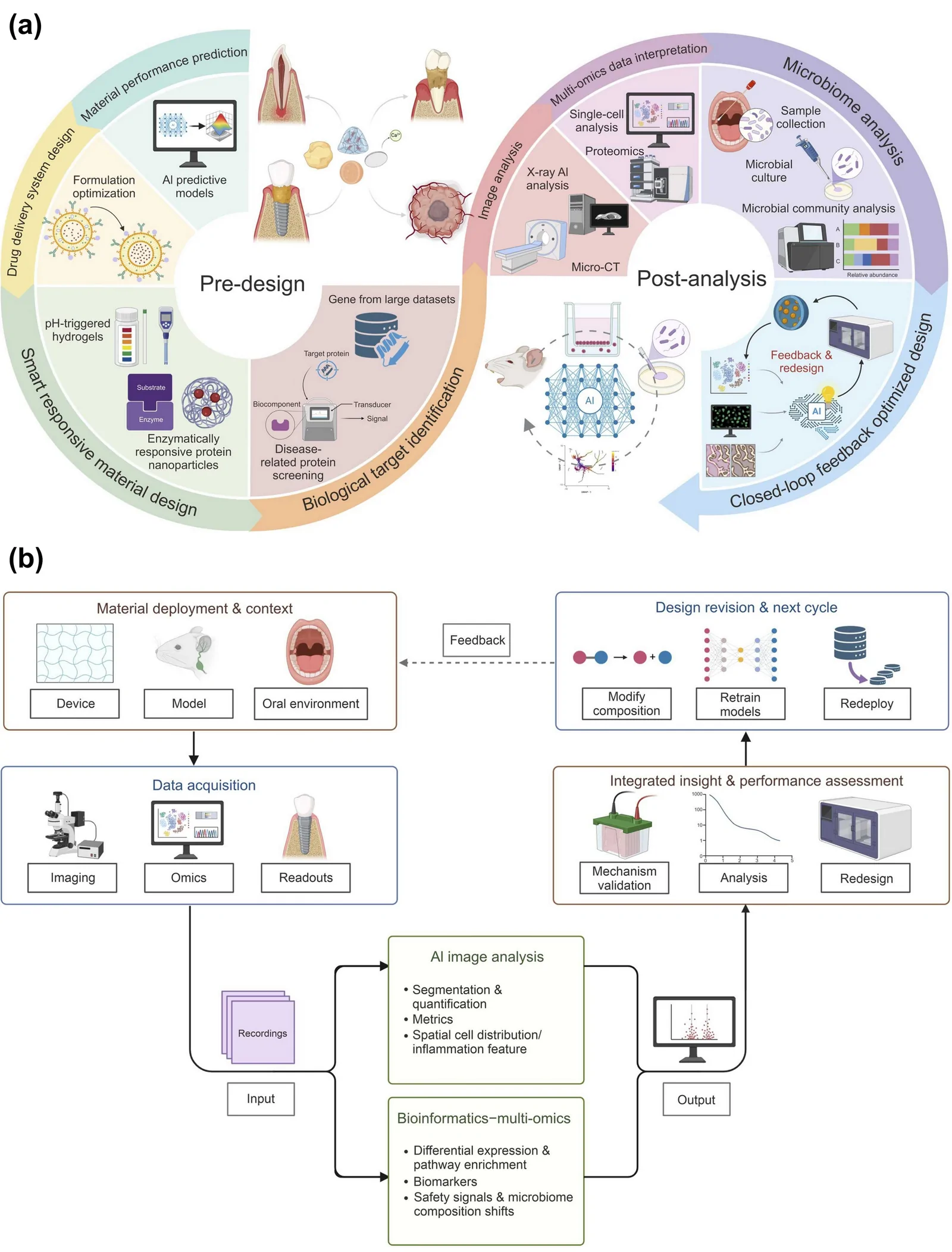
(**a**) Schematic representation of AI- and bioinformatics-based closed-loop design and validation of dental biomaterials for tissue engineering and drug delivery. (**b**) Schematic illustration of an AI/ML-based closed-loop workflow for dental biomaterials design and validation [173]. Reproduced with permission from ref. [173], Copyright 2026, ELSEVIER.

In restorative dentistry and tissue engineering, biomaterial design and implementation are difficult because of the complex interactions between biomaterials and tissues, patient-to-patient variability, and costly trial-and-error iterations. AI and bioinformatics are complementary technologies to tackle these issues [170]. In drug delivery, AI models fine-tune drug carriers (e.g., nanoparticles, hydrogels, polymer matrices) to achieve desired drug release and targeting requirements, accounting for factors such as cross-link density, particle size, and surface chemistry. Bioinformatic analyses of oral diseases reveal inflammatory or oncogenic signalling and microbial profiles that determine payload and trigger stimulus-responsive designs. These approaches enable targeted, patient-specific delivery for optimal efficacy and minimal off-target effects [171,172]. Figure 13a demonstrates this AI- and bioinformatics-optimized development process, linking target identification, design, data analysis, and refinement into a continuous loop [173]. However, there is still room for improvement. A major challenge is the lack of a large, curated dataset. For instance, there are thousands of studies on dental materials, but the data are not always consistent, and it is difficult to assemble the large datasets needed for ML [174]. This can limit the accuracy of models, as was observed in the dental composite study, where the limited size of the data set reduced the predictive power [175]. There is a multidisciplinary nature to the work: to build and interpret ML models, materials scientists, biologists, clinicians, and data scientists must work together to interpret the algorithm’s predictions and translate them into innovations [176]. Overall, bioinformatics and AI/ML provide a robust platform for next-generation biomaterials research through data-driven discovery and design [177,178,179,180]. A closed-loop AI/ML process (Figure 13b) drives dental biomaterial development through an iterative cycle of design, experimentation and refinement [173]. AI presents a promising opportunity, but it must be integrated and monitored with caution to ensure that technological developments enhance human participation in dentistry [181,182,183,184,185,186,187,188,189,190,191]. As AI continues to advance, its integration into education, research, and clinical practice offers a significant prospect to improve standards in modern dentistry.

AI may eventually support the prediction and personalization of nanotherapeutic responses by integrating multiple sources of patient-specific information, including oral microbiome profiles, disease severity, immune characteristics, lifestyle or nutritional factors, and previous treatment history. However, such predictive models remain at an early stage and are currently limited by the availability of large, standardized, and clinically representative datasets. Future progress will require longitudinal datasets, external validation across diverse patient populations, interpretable algorithms, and prospective clinical testing. Thus, AI should currently be viewed as a promising tool for future treatment stratification rather than a validated method for predicting individual responses to dental nanotherapies.

For clinical applications, additional challenges include limited dataset size, imbalance between disease categories, lack of external validation, dataset bias, variability among imaging and clinical-data acquisition protocols, and limited interpretability of complex models. Patient privacy and appropriate governance of clinical and microbiome data must also be considered. Consequently, AI-based predictions should not be regarded as clinically reliable solely on the basis of high performance within a training or internal validation dataset. Independent external validation, transparent reporting, reproducible analytical workflows, and appropriate regulatory oversight will be necessary before AI-assisted nanotherapeutic design or treatment prediction can be incorporated into routine clinical decision-making.

## 6. Future Challenges and Perspectives

Despite the notable advances in our understanding of the etiology and disease progression in dentistry, the translation of next-generation therapeutic paradigms into reliable clinical outcomes remains a significant challenge. The oral cavity represents a highly dynamic microenvironment, where constant mechanical forces, fluctuating pH, enzymatic activity, and complex microbial communities continuously test the durability and efficacy of novel interventions. Although smart biomaterials, nanotechnology, and targeted delivery strategies have presented viable alternatives to conventional antibiotics, their clinical acceptance is still constrained by multifaceted material, biological, and regulatory barriers. This segment outlines the key hurdles and future directions that require interdisciplinary solutions for sustainable clinical success.

### 6.1. Smart Drug Delivery Systems

Localized drug delivery systems are promising for managing dental infections via sustained and site-specific drug release. However, regulating gelation behavior and rheological properties, as well as predictable drug release kinetics, remains challenging. Future research should aim to develop stimuli-responsive system platforms that can respond to the dynamic oral environment to achieve controlled release of multifunctional payloads with antibacterial, anti-inflammatory, and tissue-regenerative properties while ensuring stability during storage and clinical application.

### 6.2. Multifunctional Therapeutics for Infection and Inflammation

Integrating the antimicrobial, anti-inflammatory and antioxidant properties within a single biomaterial system has potential for comprehensive disease control. The inclusion of active agents that influence oxidative stress and inflammation, along with antibacterial agents, can improve treatment efficacy, especially in complex diseases like periodontitis. Developing such multifunctional platforms will need to focus on tuning the balance of dosages, release kinetics and long-term biocompatibility.

### 6.3. Spatiotemporal Regenerative Biomaterials

Periodontal and alveolar bone regeneration requires biomaterials that can promote a sequential healing process. Composite materials, injectable hydrogels, and bioactive scaffolds with osteoinductive, proangiogenic, and stem cell-homing signals offer great potential. The challenge is to develop platforms that can deliver therapeutic agents with spatiotemporal control to ensure that antimicrobial activity is followed by sustained tissue regeneration for predictable healing.

### 6.4. Smart and Functional 3D-Printed Dental Constructs

Additive manufacturing unlocks new possibilities for customized dental implants and prosthetics; the incorporation of therapeutic effects into these constructs is in the early stages. Future developments should focus on integrating antimicrobial nanoparticles, drug-releasing matrices, and bioactive coatings within 3D-printed scaffolds while preserving mechanical strength and cytocompatibility. Controlled release in different oral environments will be essential to enable these systems to be translated into clinically safe and infection-resistant dental implants.

### 6.5. AI-Based Data-Driven Therapeutics

Artificial intelligence has the potential to significantly optimize biomaterial design, predict therapeutic outcomes, and enable personalized treatment strategies. The selection of material compositions based on patient-specific microbial profiles and disease progression patterns can be made easier with AI-driven models. Smart diagnostics combined with bioresponsive materials may lead to adaptive therapies, but data standardization, validation and ethical issues need to be overcome for clinical translation.

### 6.6. Biosafety and Long-Term Clinical Performance

The clinical translation of nanotherapeutics requires a balance between antimicrobial efficacy and long-term biological and material safety. ROS-generating systems may damage host cells and beneficial oral microorganisms when oxidative activity is excessive or poorly localized, while antibacterial implant coatings must retain their activity without compromising surface integrity, adhesion, corrosion resistance, mechanical stability, or osseointegration. These challenges are closely interconnected, as increasing antimicrobial potency should not come at the expense of host compatibility or implant functionality. Future research should therefore focus on controlled ROS generation and stable nanocoatings, followed by long-term evaluation using relevant host-cell, microbiome, mechanical, corrosion, and osseointegration models under physiologically relevant conditions. Such integrated assessment will be essential for developing nanotherapeutics that are not only effective against infection but also safe, durable, and clinically reliable.

### 6.7. Translation, Standardization, and Commercialization

While in vitro and in vivo studies have demonstrated positive results, clinical translation is limited by factors such as oral microbiota diversity, lack of testing standards, and regulatory issues. The next steps will involve the development of rigorous testing protocols, long-term biosafety and cost-effective manufacturing. Interdisciplinary approaches between material scientists, dentists and industry will be crucial to move innovations from the laboratory to real-world dental applications.

**Scheme 2:**
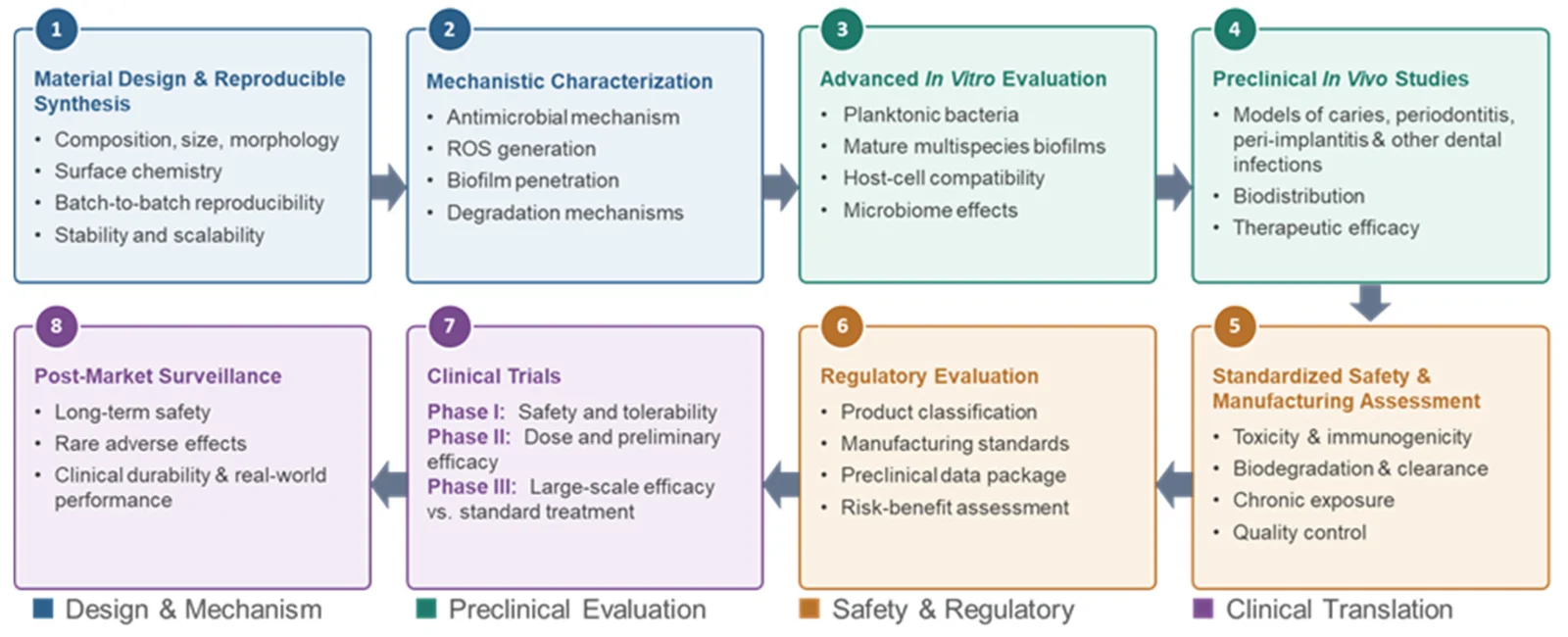
Proposed translational pathway for dental nanotherapeutics.

Scheme 2 outlines the proposed pathway for translating dental nanotherapeutics from material design and mechanistic investigation toward preclinical testing, standardized safety assessment, regulatory evaluation, and clinical trials.

Overall, the nanotherapeutic strategies discussed in this review should be regarded as emerging approaches rather than established clinical treatments. Their progression toward clinical trials will require systematic evaluation of antimicrobial efficacy in clinically relevant biofilm and tissue models, therapeutic-window determination, host-tissue and microbiome safety, long-term toxicity, reproducibility, scalable manufacturing, and regulatory compliance. Importantly, the clinical development of these systems should consider whether they provide a meaningful advantage over or complement existing dental treatments. Thus, the future clinical translation of dental nanotherapeutics will depend on progressive validation from advanced in vitro and ex vivo models to appropriate animal studies and, ultimately, well-designed human clinical trials.

## 7. Conclusions

Dental infections present a complex and multifactorial clinical challenge, primarily sustained by biofilm-associated microorganisms that exhibit increased resistance to conventional antimicrobial treatments. This review presented an in-depth overview of pathogen-targeted nanotherapeutic strategies designed to overcome these limitations by enabling localized, mechanism-driven microbial inactivation. The virulence characteristics of primary oral pathogens and their resistance within biofilm structures highlight the need for non-antibiotic treatment approaches.

Therapeutic paradigms such as photodynamic, photothermal, sonodynamic, chemodynamic, and nanozyme-based approaches demonstrate significant potential in addressing these challenges. Their ability to generate localized bactericidal effects, disrupt biofilm architecture, and minimize systemic exposure positions them as promising alternatives to traditional pharmacological therapies. Notably, the incorporation of these modalities into multifunctional material platforms, such as nanocomposite coatings, injectable hydrogels, and smart delivery systems, improves their translational significance for diverse dental applications.

While promising preclinical results have been reported, continued advances towards clinical translation will require the development of consistent evaluation protocols, long-term biosafety studies, and scalable manufacturing processes. Similarly, integrating artificial intelligence into diagnostic and material design processes may also enhance the targeting and personalization of therapeutic strategies. Overall, pathogen-targeted nanotherapeutics show promise in preclinical models to deliver more targeted, sustained and clinically versatile solutions for dental infection management.

### Literature Search and Study Selection

A structured literature search was conducted to identify studies relevant to nanomaterial-based therapeutic strategies for bacterial and non-bacterial dental infections. The literature was retrieved primarily from Web of Science, Scopus, PubMed, and Google Scholar using combinations of keywords including “dental infection”, “oral biofilm”, “periodontitis”, “peri-implantitis”, “antimicrobial nanomaterials”, “photodynamic therapy”, “photothermal therapy”, “chemodynamic therapy”, “sonodynamic therapy”, “nanozyme”, “reactive oxygen species”, “gas therapy”, and “nanoparticle-based drug delivery”. Priority was given to peer-reviewed original studies and recent review articles that directly addressed the therapeutic mechanisms, antimicrobial performance, biosafety, or translational potential of these approaches.

Studies were included when they provided relevant evidence on nanotherapeutic strategies for oral pathogens, dental biofilms, or clinically relevant dental disease models. Articles were excluded when they were unrelated to oral or dental applications, provided insufficient information regarding the nanomaterial or therapeutic mechanism, or focused exclusively on non-therapeutic diagnostic applications. As this article is intended as a narrative review, the search strategy was structured to ensure broad and balanced coverage rather than to perform a formal systematic review or meta-analysis.

## Figures and Tables

**Table 1:** Relative analysis of antibacterial and biofilm-targeted therapeutic approaches for dental healthcare management.

Therapeutic Strategies	Mechanism	Advantages	Disadvantages	Clinical Potential
**PTT**	Non-radiative relaxation generating localized hyperthermia	Rapid localized heating kills bacteria and disrupts biofilms. Non-antibiotic approach reduces risk of resistance. It can be integrated with nanomaterials for targeted action.	Risk of thermal damage to surrounding tissues. Limited NIR-light penetration depth. Requires precise energy control.	Promising for localized dental infections; still at preclinical and early translational stages.
**PDT**	Type II photochemical reaction	ROS generation inactivates bacteria and biofilms. Effective against resistant strains. Minimally invasive and repeatable.	Dependent on oxygen availability and light penetration. Photosensitizers may cause instability or side effects. Shallow tissue penetration.	Already applied in dentistry (e.g., periodontal treatment); requires optimization for broader clinical use.
**CDT**	Fenton or Fenton-like reaction	Produces ROS via catalytic reactions in situ. Exploits the acidic, H_2_O_2_-rich microenvironment of infections. Selective antibacterial action.	Limited by endogenous H_2_O_2_ levels. Excess ROS may cause cytotoxicity. Less effective in neutral conditions.	Strong experimental data; clinical translation is still at the proof-of-concept stage.
**SDT**	US cavitation	Deep tissue penetration by US. Effective ROS production against biofilms. Non-invasive and site-targeted.	Relies on efficient delivery of sonosensitizers. Risks of cavitation or heating. Limited clinical data in dentistry.	Potentially valuable for deep-seated infections; currently preclinical.
**Nanozyme-based Antibacterial Therapy**	Enzyme-like catalytic reactions	Stable enzyme-mimicking activity. Multifunctional (antibacterial, antioxidant, regenerative). Effective in biofilm disruption.	Long-term biosafety and clearance are uncertain. Dependent on the infection microenvironment. Large-scale reproducibility challenges.	High translational potential; more preclinical successes, but limited clinical validation.

**Table 2:** Comprehensive analysis of nanotherapeutic strategies for dental infections based on mechanism, disease model, targeted disease and organisms, host compatibility, clinical evidence, and limitations.

Therapeutic Strategy	Target Disease	Target Organism	Model	Nanomaterial/Formulation	Stimulus	Mechanism	Host Compatibility	Clinical Evidence	Major Limitation	Ref.
**PDT**	Dental caries/dentinal infection	*S. mutans*, *L. acidophilus*	Planktonic; monospecies biofilms; carious human dentin	Toluidine Blue O	635 ± 10 nm LED, 31.5 J cm^−2^, 60 s	Type-II photochemical ROS generation	Not the primary focus; tissue-deep effects limited	No	Reduced efficacy in deeper dentinal caries/tubules; oxygen and light penetration limitations	[142]
**PDT**	Dental caries/oral biofilms	*S. mutans*, *C. albicans*	Monospecies biofilms	Methylene-blue-loaded Pluronic F127 polymeric micelles	660 nm light	Photosensitizer-generated ROS with enhanced biofilm penetration/release	Favorable formulation characteristics; no clinical safety evidence	No	Clinical applicability remains unvalidated; penetration and irradiation conditions require optimization	[143]
**PDT**	Caries-affected dentin	*S. mutans*	Ex vivo infected human caries-affected dentin	Phycocyanin-loaded Ag nanoparticles	PDT/Nd:YAG laser	ROS generation enhanced by Ag nanoplatform	Maintained/highest shear bond strength (17.23 ± 1.45 MPa)	No	Ex vivo model; no long-term tissue or clinical evidence	[144]
**PDT**	Periodontitis	Subgingival periodontal bacteria, including *P. gingivalis*	Human periodontal pockets; clinical study	Chitosan-coated ICG-encapsulated nanoparticles	810 ± 20 nm transgingival laser	NIR-activated PDT; bacterial targeting by positively charged chitosan coating	No pain reported; one participant reported discomfort; no visible laser-induced tissue damage	Yes—randomized clinical study, n = 40	Bactericidal effect was lower and shorter-lived than predicted from laboratory studies	[145]
**PDT/PTT**	*P. gingivalis* oral infection/periodontitis	*P. gingivalis*	In vitro bacterial infection; golden-hamster oral infection model	Oxyhemoglobin-IR820 nanophotosensitizer	808 nm NIR light	Combined photothermal heating + oxygen-assisted PDT; targeted uptake by *P. gingivalis*	Favorable in vivo safety reported; oxygen donor addresses anaerobic PDT limitation	No	Complex multifunctionality; clinical PDT/PTT parameters and long-term safety remain to be established	[146]
**PTT**	Periodontitis	*P. gingivalis*, *F. nucleatum*	Planktonic bacterial cultures; rat periodontitis model	Baicalein-loaded mesoporous Prussian blue (MPB-BA)	808 nm NIR; ~50–55 °C therapeutic range	Photothermal membrane disruption + baicalein-mediated antioxidant/anti-inflammatory modulation	Anti-inflammatory and antioxidant effects; favorable in vivo response reported	No	High temperatures can cause tissue injury; optimal photothermal window must be controlled	[147]
**PTT**	Periodontitis	*S. aureus*, *E. coli*; oral plaque biofilm	In vitro bacterial/biofilm models; rat periodontitis	EGCG-loaded Au nanoparticle hydrogel (E-Au@H)	NIR irradiation; ~50.7 °C after 5 min	Photothermal antibacterial action + NIR-controlled EGCG release + regeneration	Promoted angiogenesis and bone regeneration	No	Requires controlled temperature and sufficient NIR penetration; formulation complexity	[148]
**PTT**	Periodontitis	*P. gingivalis*	Established biofilm; rat periodontitis	Fe_3_O_4_/ZnO/ε-polylysine (FZE) nanospheres	808 nm NIR, 1.0 W cm^−2^ photothermal heating	Mild photothermal disruption of dense biofilm facilitates ZnO/EPL penetration and bacterial killing	Acceptable in vitro and in vivo biosafety reported	No	Still preclinical; heat transfer and NIR penetration require optimization	[149]
**PTT**	Dental caries/secondary caries	*S. mutans*	Planktonic cultures; 24 h bacteria grown on dental resin discs	Au nanoshell-loaded dental resin	660 nm laser; 95.5 J cm^−2^	Photothermal heating from Au nanoshells	No significant adverse material-polymerization effect reported; tissue safety not established	No	Heat transfer through cured resin and absence of nanoparticles at surface substantially limited efficacy	[150]
**SDT**	Dental caries	*S. mutans*	Planktonic bacterial culture	Nanomicelle curcumin (NM@Cu)	Ultrasound irradiation	Ultrasound-activated sonosensitizer generating ROS	NM@Cur showed lower cytotoxic/apoptotic effects toward human gingival fibroblasts than free curcumin	No	Requires optimization of acoustic parameters and validation in clinically representative biofilms	[151]
**SDT**	Dental caries	*S. mutans*	**Preformed biofilm on enamel slabs**	ZnO/TiO_2_ nanoparticle combination	Ultrasound; 0.75 W cm^−2^; particle-specific sub-significant reduction doses	Ultrasound-activated ROS production and nanoparticle-assisted sonodynamic activity	Cytotoxicity/long-term tissue safety not comprehensively established	No	Acoustic frequency, duty cycle, cavitation and tissue penetration require further validation	[152]
**CDT**	Dental caries	*S. mutans*	EPS-rich biofilm; acidic oral-microenvironment model; rat caries model	Cu-doped ZnO_2_ loaded with Rose Bengal (ZnO_2_-Cu@RB)	Acidic biofilm microenvironment; endogenous proton-triggered H_2_O_2_ generation	Self-supplied H_2_O_2_+ Cu-mediated Fenton-like reaction generating ·OH	No harmful effects reported in animal model	No	Clinical translation depends on control of peroxide generation, degradation products and metal exposure	[153]

## Data Availability

No new data were created or analyzed in this study. Data sharing is not applicable to this article.

## Abbreviations

PDT	Photodynamic therapy
PTT	Photothermal therapy
CDT	Chemodynamic therapy
SDT	Sonodynamic therapy
ROS	Reactive oxygen species
EPS	extracellular polymeric substance
*S. mutans*	*Streptococcus mutans*
*P. gingivalis*	*Porphyromonas gingivalis*
*C. albicans*	*Candida albicans*
*E. coli*	*Escherichia coli*
•OH	hydroxyl radicals
US	ultrasound
ML	Machine learning
AI	artificial intelligence
PTAs	photothermal agents
NIR	near-infrared
PSs	photosensitizers
AIE	aggregation-induced emission
